# A high-resolution data set of fatty acid-binding protein structures. II. Crystallographic overview, ligand classes and binding pose

**DOI:** 10.1107/S2059798325005728

**Published:** 2025-07-28

**Authors:** Andreas Ehler, Joerg Benz, Markus G. Rudolph

**Affiliations:** a Therapeutic Modalities, Innovation Center Basel F. Hoffmann-La Roche Grenzacherstrasse 124 4070Basel Switzerland; University of Oxford, United Kingdom

**Keywords:** isoform specificity, crystal pathologies, ligand binding, fortuitous SAD phasing

## Abstract

Fatty acid-binding protein isoforms 4 and 5 are potential diabetes and atherosclerosis targets. During a drug-design program aiming at dual isoform-specific FABP4/5 inhibitors with little or no affinity for FABP3, a set of crystal structures with a median resolution of 1.2 Å was generated. The chemical space of the ligands covers various series in which the carboxylate and aliphatic groups of the natural fatty-acid ligands have been replaced by other moieties. A summary of binding modes of the chemical series is also given with respect to how isoform specificity was achieved. Additionally, several bromine-containing ligands were identified that allowed SAD phasing, yielding an independent experimental confirmation of their chemical composition.

## Abstract 2

A high-quality set of 229 crystal structures of various isoforms of human and mouse fatty acid-binding proteins in apo and ligand-bound forms is presented. Many ligands have associated affinity data, which may help in the development of affinity- and pose-predicting algorithms.

## 1. Introduction

**Figure 1:**
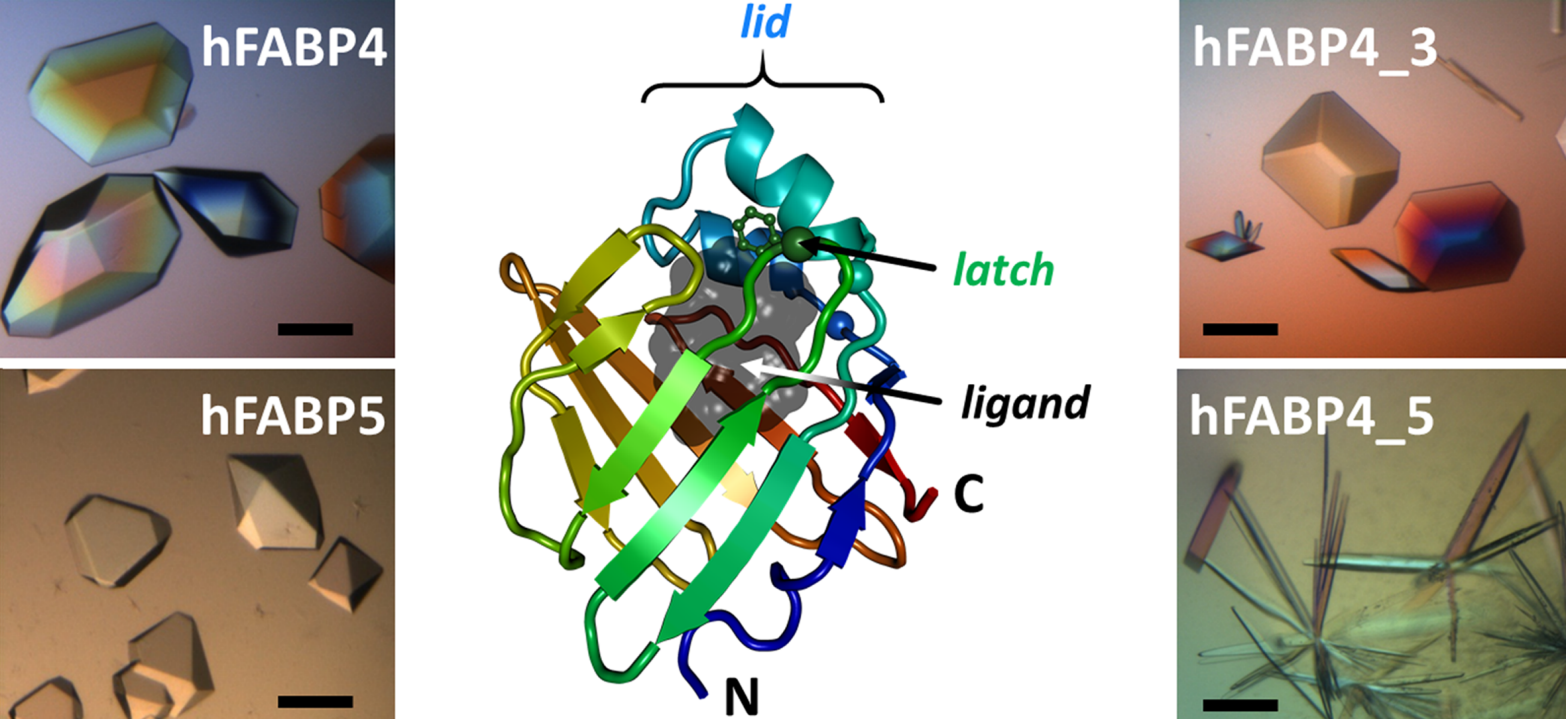
Crystals and architecture of FABP. The ribbon representation displays the ten-stranded β-barrel fold of hFABP4 (PDB entry 7g0n, ligand-free, obtained from delipidated hFABP4) with the chain colored in a rainbow from the N-terminus to the C-terminus (marked). The cavity available for fatty acid or other ligand binding is shown as a gray volume of 540 Å^3^ (1.4 Å probe radius). Fig. 7 ▸(*a*) shows FABP4_5 in a similar orientation with a fatty acid bound. The scale bars for the different crystal forms are ∼0.4 mm.

Fatty acid-binding proteins (FABPs) belong to the calycin superfamily of proteins, sharing a similar overall structure with a ten-stranded β-barrel that encloses a large interior cavity for fatty-acid binding. Access of ligands to the binding site is regulated by a small α-helical subdomain (lid) that can be fixed by interaction with an opposing latch (Fig. 1 ▸). The different FABP isoforms regulate the uptake, metabolism and intracellular trafficking of fatty acids (reviewed in Storch & McDermott, 2009 ▸; Supplementary Table S1).

Based on epidemiological studies and animal knockout models, the FABP4 and FABP5 isoforms were identified as potential diabetes and atherosclerosis targets (reviewed in Furuhashi & Hotamisligil, 2008 ▸). Both isoforms are produced, among others, in adipocytes and macrophages and share a sequence identity and similarity of 54% and 72%, respectively. Deletion of the *FAB4* gene in mice reduces hepatic steatosis, improves glucose tolerance and increases insulin sensitivity. At the same time, in several genetic models, inflammation and atherosclerotic lesion size is reduced. In humans, *FABP4* haplo-insufficiency is associated with a decreased risk of type 2 diabetes and cardiovascular disease. Increased concentrations of FABP4 have been detected in diabetic patients, leading to obesity and atherosclerotic lesions (reviewed in Furuhashi & Hotamisligil, 2008 ▸). However, it has also been found that a lack of FABP4 can be functionally complemented by FABP5, which is upregulated in the adipose tissue of *FABP4*^−/−^ mice (Shaughnessy *et al.*, 2000 ▸; Hertzel *et al.*, 2006 ▸). A double knockout of the *FABP4* and *FABP5* genes in mice displayed a stronger phenotype than the individual knockouts, including protection from diet-induced obesity, insulin resistance, type 2 diabetes and fatty liver disease (Maeda *et al.*, 2005 ▸). Thus, an efficient inhibitor for these indications would have to bind to both FABP isoforms with high affinity. FABP3, on the other hand, is prominent in the heart, and *FABP3* knockout mice display improved cardiac function and decreased cardiac myocyte apoptosis after myocardial infarction (Zhuang *et al.*, 2019 ▸). To prevent unwanted effects on cardiac energy metabolism, an optimal FABP inhibitor would display dual activity against FABP4 and FABP5 while not binding to FABP3. As a further requirement, the testes-specific isoform FABP9 might be included, as male *FABP9* knockout mice display abnormal sperm morphology (Selvaraj *et al.*, 2010 ▸). Taken together, while partial inhibition of FABP9 may be tolerated, a high degree of selectivity against FABP3 appears paramount to avoid any potential adverse cardiac effects.

Human FABP isoforms hFABP4 and hFABP5 crystallize reproducibly, and apo or fatty acid-bound crystals can be soaked with a variety of ligands. The hFABP4 construct proved to be highly reproducible in crystallization and very tolerant to high ligand and DMSO concentrations during soaking, while retaining diffraction to high resolution. As an alternative to individual crystallization, structural information on FABP isoforms may be gained by mimicking the binding site of the isoform in the context of hFABP4. Octuple variants of hFABP4 changed the binding sites to those of isoforms 3 and 5, termed hFABP4_3 and hFABP4_5, while retaining the favorable surface properties of hFABP4 for crystallization.

These efforts led to a set of 229 crystal structures, 216 of which have a ligand bound. Several of the structures are associated with additional affinity data. In total, 75 structures contain a ligand for which IC_50_ values were measured for hFABP isoforms 4 and 5, while for 50 structures IC_50_ values are available for hFABP isoforms 3, 4 and 5. The IC_50_ values of all disclosed FABP inhibitors have been determined using the same biochemical assay, thus the data are intrinsically consistent and should serve as a rich resource for the training of machine-learning or other algorithms for the prediction of ligand-binding poses and relative binding affinities. Affinity estimation in particular has proven to be notoriously difficult to achieve and is always target-centered (Shortridge *et al.*, 2008 ▸; Leidner *et al.*, 2019 ▸; Parks *et al.*, 2020 ▸). We recently developed a docking workflow based on the piecewise linear potential scoring function to generate physically plausible ligand poses and compared prediction models in different scenarios encountered in drug discovery for the target PDE10 (Tosstorff *et al.*, 2022 ▸). The prediction models derived from that data set are relevant for that target only, which is a standard situation in lead optimization, but unsatisfactory in general. To generalize the predictions to other targets, there is a need to publish diverse sets of high-quality protein–ligand co-crystal structures plus accompanying affinity data, to which this FABP data set shall be a contribution.

## 2. Materials and methods

### 2.1. FABP isoform and variant purification

Coding sequences for all FABP isoforms (human 1, 3, 4, 5 and 9 and mouse 4 and 5) and variants (human FABP4_3 and FABP4_5) were cloned using Bpu1102I and NdeI into pET-15b coding for an N-terminal hexahistidine tag followed by a thrombin recognition site. The proteins were produced in *Escherichia coli* BL21(DE3) cells and the cells were disrupted by a French press in 50 m*M* Tris–HCl pH 8.0, 300 m*M* NaCl (buffer *A*) including protease inhibitor (Roche Complete). After the addition of 5 m*M* diisopropyl fluoro­phosphate (DIFP) and 2 m*M* MgCl_2_, the crude extract was cleared by centrifugation at 40 000*g* at 4°C for 30 min. The supernatant was passed through a 0.22 µm sterile filter and applied onto a 5 mL HisTrap HP (GE Healthcare, catalogue No. 17-5248-01) Ni^2+^–NTA affinity column equilibrated in buffer *A*. The protein was eluted with a gradient to 500 m*M* imidazole in buffer *A* over five column volumes (25 mL). Pooled fractions were supplemented with 2.5 m*M* CaCl_2_ and 1000 U thrombin (GE Healthcare) and dialyzed at 4°C overnight in a Slide-a-Lyzer (3–12 mL, 3000 molecular-mass cutoff) against 25 m*M* Tris–HCl pH 7.5, 100 m*M* NaCl. The flowthrough from a second round of Ni^2+^–NTA affinity chromatography in buffer *A* was collected, concentrated and applied onto a Superdex 75 10/300 gel-permeation column equilibrated in 25 m*M* Tris–HCl pH 7.5, 100 m*M* NaCl, 10% glycerol. FABP4_3 is the octuple variant V24L, M41T, I52L, S54T, I105L, V116L, C118L,S125C and FABP4_5 is the octuple variant V24L, V33M, A34G, M41C, S54T, F58L, H94Q, S125C (FABP4 numbering). These variants convert the binding site of human FABP4 into those of the FABP3 and FABP5 isoforms while keeping the FABP4 surface residues for ready crystallization and facile soaking.

### 2.2. Crystallography

FABP crystals were obtained in several crystal forms in a sitting-drop vapor-diffusion setup at 295 K from 10–30 mg mL^−1^ protein solution mixed 1:1 with reservoir, as summarized in Supplementary Table S2. Ligands were dissolved at 200 m*M*in DMSO and crystals were soaked at a final concentration of 30% DMSO (60 m*M* ligand). If needed, crystals were cryoprotected (as listed in Supplementary Table S2) before vitrification in liquid nitrogen. Diffraction data were collected at 100 K over a range of 180° with an oscillation range of 0.25° or 0.5° at a wavelength of 0.7, 0.8, 0.9 or 1.0 Å, depending on the high-resolution limit of test images. Since phasing of the data was not an issue and the identity of the ligands was known (or so we thought), no adjustment of the data-collection wavelength to maximize anomalous signal from FABP crystals soaked with halogen-containing ligands was made. Data were collected on beamline PXII of the Swiss Light Source using a MARmosaic 225 mm CCD or PILATUS2 6M detector, and were integrated and scaled with *XDS* and *XSCALE* (Kabsch, 2010 ▸), respectively, using a simple in-house data-processing pipeline. Initial phases were obtained by molecular replacement with *Phaser* (McCoy *et al.*, 2007 ▸) using an in-house FABP4 model, and models were rebuilt in *Coot* (Casañal *et al.*, 2020 ▸) and refined with *REFMAC*5 (Murshudov *et al.*, 2011 ▸). Individual anisotropic *B* values were refined for data sets exceeding 1.2 Å resolution. The high-resolution limit at the time of data collection (2010–2013) was placed at 1–2 *I*/σ in the outer shell, which nowadays would roughly correspond to a CC_1/2_ of between 0.3 and 0.5. Ligand restraints were generated using *Grade* (Global Phasing, UK) with *Mogul*-extracted data statistics from the CCDC (Bruno *et al.*, 2002 ▸) and, if necessary, adjusted based on visual inspection of unbiased *F*_o_ − *F*_c_ omit electron density after rebuilding and refinement of the protein part of the model. For display purposes, *F*_o_ − *F*_c_ maps for figures were calculated from final models after removal of the ligand, refinement of the incomplete model to convergence using the same protocol and contouring at the r.m.s.d. values indicated in the individual figures. A summary of all crystal structures is given in Supplementary Excel File S1. Individual crystallographic statistics, including translation–libration–screw (TLS) and noncrystallographic symmetry (NCS) parameters, if used, are placed in the headers of the coordinate files, which have been released in the PDB along with the structure factors.

Bromine-containing ligands offered the unique opportunity for SAD phasing, which was successful in five out of nine cases (Table 2). Starting from the unmerged reflections in XDS_ASCII.HKL, *HKL*2*MAP *(Pape & Schneider, 2004 ▸) was used to prepare the data for SAD phasing. 1000 *SHELXD* (Schneider & Sheldrick, 2002 ▸) trials were run, followed by 200 cycles of density modification and ten cycles of model building in *SHELXE* (Thorn & Sheldrick, 2013 ▸). An overestimated solvent content of 50% was used to aid phasing by emphasizing solvent flattening. For the unsuccessful SAD cases, another scaling strategy, *i.e.AIMLESS* (Evans & Murshudov, 2013 ▸) followed by *STARANISO* (Global Phasing, UK) and high-resolution estimation using CC_1/2_ > 0.3 in the high-resolution shell, was used (Karplus & Diederichs, 2012 ▸), but this approach did not change the negative outcome. Also unsuccessful were attempts at SIRAS phasing using the ultrahigh-resolution data set (PDB entry 7fxw; 0.88 Å resolution) as the native. Throughout the text, PDB entries and the ligands they contain are simply labeled by their four-character PDB codes. Structure figures were prepared with *PyMOL* (Schrödinger) and the p*K*_a_ values of the ligands were calculated with A*DMET Predictor* (Fraczkiewicz *et al.*, 2024 ▸).

### 2.3. IC_50_ determination

IC_50_ values of compounds for FABP isoforms were determined using a Förster resonance energy transfer (FRET) assay with Tb^3+^ as the donor and BODIPY as the acceptor, as described in a technical handbook (https://resources.revvity.com/pdfs/gde-htrf-technical-booklet.pdf). A BODIPY-labeled C_11_ fatty acid (Invitrogen, catalogue No. D3862) was incubated with 50 n*M* His_6_-tagged FABP in 25 m*M* Tris–HCl pH 7.5, 0.4 mg mL^−1^ γ-globulin (Sigma), 1 m*M* DTT and 0.012–0.077% NP-40 depending on the FABP isoform (0.012% for hFABP4 and hFABP5, 0.032% for hFABP1 and mFABP4, 0.077% for hFABP3 and 0.051% for mFABP5). The final concentrations of the BODIPY-labeled C_11_ fatty acid were 125 n*M* (hFABP1, hFABP4 and hFABP5), 300 n*M* (mFABP5) or 600 n*M* (hFABP3). A Tb^3+^-bound anti-His_6_ antibody (Columbia Biosciences) in 25 m*M* Tris–HCl pH 7.5, 0.4 mg mL^−1^ γ-globulin was added at a concentration of 4 n*M*. Tb^3+^ fluorescence was excited at 488 nm and FRET was recorded at 520 nm with a bandwidth of 10 nm. The decrease in FRET signal as a function of compound concentration between 0.003 and 50 000 n*M* was measured by an IOM NanoScan FLT plate reader after an incubation time of 30 min. Compounds were prepared as 10 m*M* DMSO stock solutions. The binary equilibrium dissociation constants (*K*_d_) of the BODIPY-labeled C_11_ fatty acid are 115, 122, 38, 1305, 87 and 611 n*M* for hFABP4, hFABP5, hFABP1, hFABP3, mFABP4 and mFABP5, respectively. *K*_i_ values were calculated from IC_50_ according to
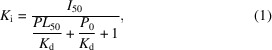
where *I*_50_ and *L*_50_ are the free concentrations of the inhibitor and the BODIPY-labeled C_11_ fatty acid, respectively, at 50% inhibition. *P*_0_ is the free concentration of FABP in the absence of inhibitor, which may be calculated from the solution of the quadratic equation describing the equilibrium characterized by *K*_d_. *I*_50_, *L*_50_ and *P*_0_ are further detailed in Burnett *et al.* (2007 ▸).

## 3. Results and discussion

### 3.1. Overview of the crystallographic data set

**Figure 2:**
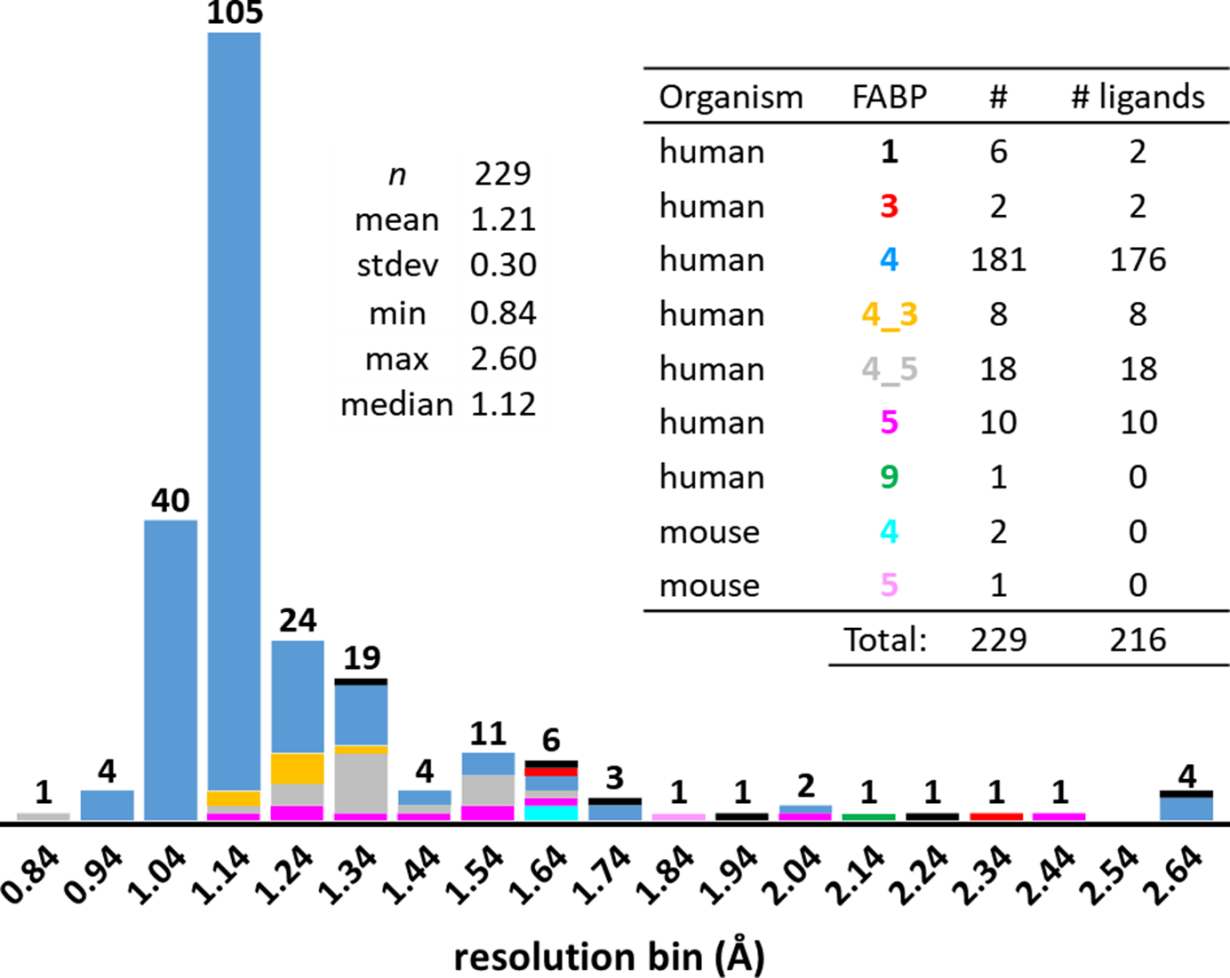
Stacked histogram of the resolution and isoform distribution of FABP structures. The color code for the isoforms is given in the inset table. The hFABP4_5 structure 7g0z (gray, far left) has a resolution of 0.84 Å, setting the bin boundary. Four structures, all of hFABP4 (blue), have resolutions of >0.84 and ≤0.94 Å *etc*. The four structures with the worst resolutions of >2.54 and ≤2.6 Å are three hFABP4 structures and one hFABP1 structure (black). The mean and median resolutions of the structures in the set are 1.21 and 1.12 Å, respectively.

The FABP data set consists of 229 crystal structures, 216 of which contain one of 187 unique ligands (Fig. 2 ▸). Six further structures (5edb, 5edc, 5hz5, 5hz6, 5hz8 and 5hz9) have been described by us previously (Kuhn *et al.*, 2016 ▸). The FABP constructs used in this study crystallized in 23 different forms (Supplementary Table S2), although some are isomorphous but contain a different FABP construct. Some of the FABP crystal forms have been described previously (see below).

Mouse and human FABP4 share 91.5/95.3% identity/similarity and mouse and human FABP5 share 80.5/93.2% identity/similarity, with some of the substitutions being in the lipid-binding site. The differences in the fatty acid-binding sites appeared to be large enough to warrant separate structure determinations. Mouse FABP4 and mouse FABP5 crystals were grown to help answer questions from studies in animal models, should they occur, but ultimately such ligand-bound structures were never needed. Two apo mFABP4 and a single mFABP5 structure were determined. The two crystal forms for mFABP4 found here are isomorphous to those published previously. 7fwt is an apo mFABP4 structure isomorphous to 2qm9 (2.3 Å; Gillilan *et al.*, 2007 ▸) and 2ans (2.5 Å; Ory & Banaszak, 1999 ▸), but now available at a resolution of ∼1.6 Å. The apo mFABP4 structure 7fzg is isomorphous to 2q9s (2.3 Å; Gillilan *et al.*, 2007 ▸), 3jsq (2.3 Å; Hellberg *et al.*, 2010 ▸) and 3hk1 (1.7 Å; Hertzel *et al.*, 2009 ▸), but now available at a resolution of 1.5 Å. Four more mFABP4 crystal structures in other crystal forms are known but these forms are not represented in our data set, *i.e.*1alb (2.5 Å; Xu *et al.*, 1992 ▸), 1lib, 1lid and 1lif (1.7, 1.6 and 1.6 Å resolution, respectively; Xu *et al.*, 1993 ▸). The 1.8 Å resolution apo mFABP5 structure 7fyw is twinned (effective resolution 2.2 Å; see below) and adds yet another crystal form to the current set of lipid-bound mFABP5 structures (4azn, 4azo, 4azp and 4azq at 2.0–2.5 Å resolution; Sanson *et al.*, 2014 ▸). Furthermore, testes-specific human FABP9 was determined in a new hexagonal space group compared with the previously released tetragonal form (4a60; J. R. C. Muniz, W. Kiyani, L. Shrestha, D. S. Froese, T. Krojer, M. Vollmar, C. H. Arrowsmith, A. M. Edwards, J. Weigelt, C. Bountra, F. von Delft & W. W. Yue, unpublished work). However, additional hFABP9 structures were not determined as this isoform was finally not included as a counter-target, *i.e.* a protein not to be inhibited, in the ligand design.

### 3.2. Presence of twinning and pseudo-translation in the data set

Of the 23 different crystal forms in the data set, three are tetragonal, one is hexagonal and three are cubic (Supplementary Table S2). All of these could support merohedral twinning, but analysis of the second moments 〈*I*^2^〉/〈*I*〉^2^ in intensities *I*, the standard deviation |*E*^2^ − 1| of the mean normalized structure-factor amplitude *E* and the local *L* values *L* = (*I*_1_ − *I*_2_)/(*I*_1_ + *I*_2_) of reflections *I*_1_ and *I*_2_ that are located close in reciprocal space (Padilla & Yeates, 2003 ▸) did not indicate twinning in these data. By contrast, two cases of pseudo-merohedral twinning were observed. Structures 7g0w (apo hFABP1) and 7fyw (apo mFABP5) both crystallized in space group *P*2_1_ with the β angle close to 90°, emulating primitive orthorhombic symmetry with twin law (−*h*, −*k*, *l*). In structure 7g0w, the indicators were clear about the twinning, with the second moment 〈*I*^2^〉/〈*I*〉^2^ = 1.67 (no twin, 2.0; perfect twin, 1.5), |*E*^2^ − 1| = 0.59 (no twin, 0.74; perfect twin, 0.54) and *L* = 0.39 (no twin, 0.5; perfect twin, 0.2). However, in structure 7fyw the presence of twinning was less clearly indicated, with the second moment 〈*I*^2^〉/〈*I*〉^2^ = 2.3, |*E*^2^ − 1| = 0.75 and *L* = 0.46. A self-Patterson map showed the presence of a peak at fractional coordinates (0.8, 0.5, 0.1) with 23% of the height of the origin. Together with anisotropy in the diffraction data, this pseudo-translation led to a slightly elevated second moment and to an unsuspicious |*E*^2^ − 1|. Only the slightly reduced *L* value indicated the presence of twinning, highlighting the usefulness of *L* as a twinning indicator, even in the presence of crystal pathologies such as pseudo-translation and anisotropy, which change the intensities and hence influence the moments and *E*. Also, merging of the data in *P*222 and molecular replacement in all primitive orthorhombic space groups and settings invariably led to clashes. The effective resolution due to twinning in this structure is about 2.2 Å. Masking of pseudo-merohedral twinning by pseudo-translation is rare but has precedents (Rudolph *et al.*, 2004 ▸; Lebedev *et al.*, 2006 ▸; Zwart *et al.*, 2008 ▸).

On the other hand, some crystal settings, both primitive monoclinic, that in principle allow pseudo-merohedral twinning fortunately did not display abnormal intensity statistics. These are the hFABP1 structures 7g1x and 7g00. In structure 7g1x (apo) the cell constants *a* = 58.7, *b* = 34.9, *c* = 59.3 Å, β = 119.2° are almost hexagonal and approximately follow the relation *c*·cosβ = −*a*/2, which is a second way for primitive monoclinic crystals to twin and emulate *C*-centered ortho­rhombic (Rudolph *et al.*, 2004 ▸). No twin refinement was necessary for these diffraction data. Structure 7g00 in complex with a cyclopentene-1-carboxylic acid is an example of a primitive monoclinic crystal with β ≃ 90° and the presence of a strong pseudo-translation vector with height 66% of the origin peak at (0.5, 0.5, 0). The data merge in primitive orthorhombic symmetry, and a well packed molecular-replacement solution with eight molecules in the asymmetric unit may be found. However, refinement of this solution failed, such that the symmetry had to be dropped to primitive monoclinic with β = 90.1°.

### 3.3. Crystal systems best suited for ligand soaking

Most of the ligand-bound structures are from just two orthorhombic crystal forms with a single FABP molecule in the asymmetric unit (Table 1 ▸) that withstood up to 50% DMSO during soaking. Extreme organic solvent tolerance has been observed by others for FABP3, crystals of which were soaked with 50% DMSO ligand solutions (7fbm, 7euv and 7euw; S. Sugiyama, K. Kakinouchi, S. Matsuoka, H. Tsuchikawa, M. Sonoyama, Y. Inoue, F. Hayashi & M. Murata, unpublished work). Here, we used soaking solutions at final concentrations of 60 m*M* ligand and 30% DMSO overnight, which did not appear to degrade the diffraction properties of the crystals.

**Figure 3:**
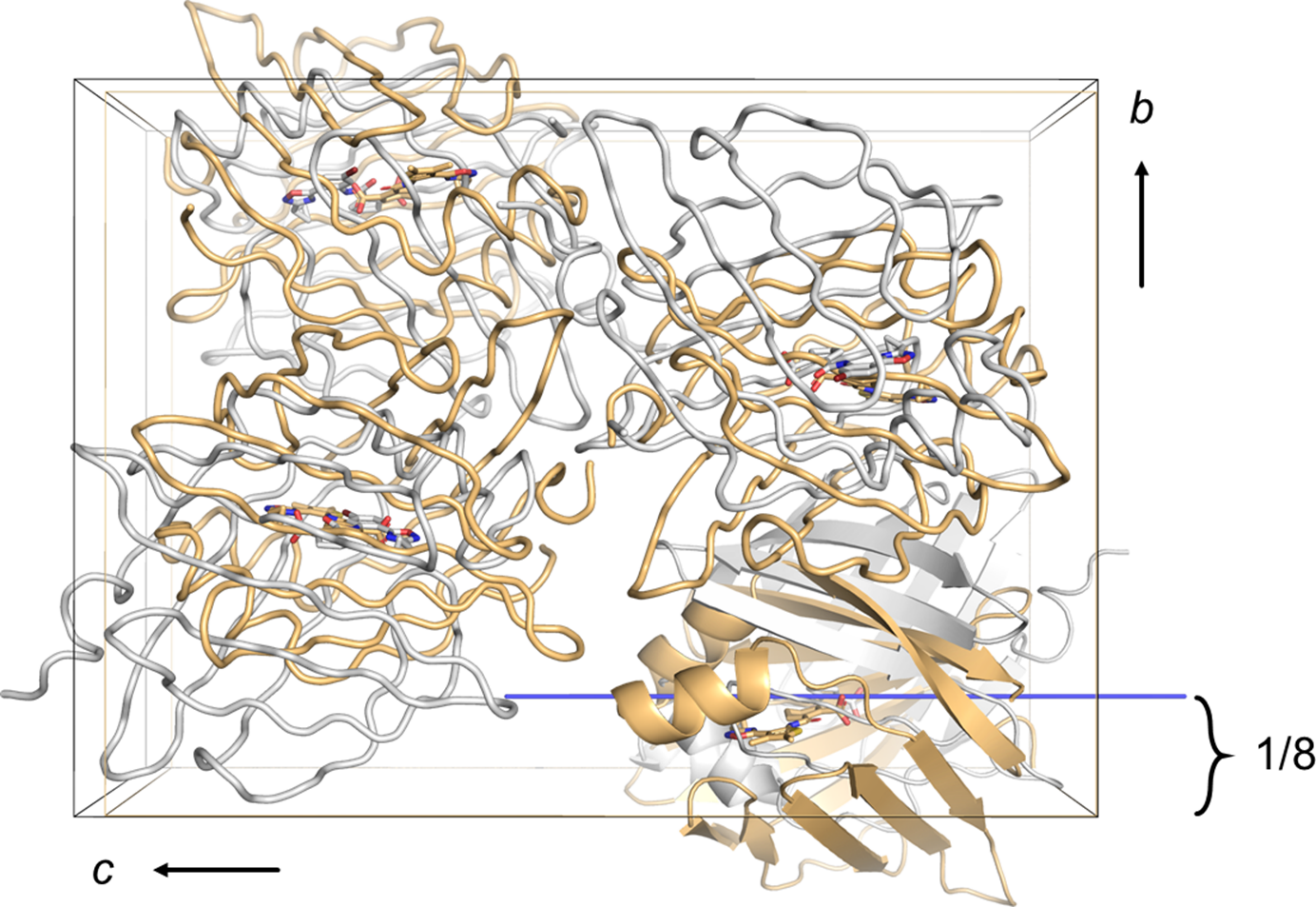
Two different crystal forms of FABP4 with quite similar cell dimensions have different packing in different space groups. Gray: 7fzw, hFABP4 in space group 19 or *P*2_1_2_1_2_1_. Sand: 7g0o, hFABP4_5 in space group 18 (setting 3018 with unique axis *a*, *i.e.P*22_1_2_1_). The two asymmetric units are related by a roughly 150° rotation about an axis that is near-parallel to the *c* axis and located at ∼1/8 along the *b* axis (blue). Molecules displayed as tubes are symmetry equivalents.

Both of these crystal forms are suitable for soaking and diffracted equally well. The two crystal forms have very similar cell dimensions of ∼32, 53 and 74 Å, but are not isomorphous because they belong to different space groups: the short axis may be a screw axis or not, leading to space groups 19 or 18. The axis order in Table 1 ▸ was chosen as space-group setting 3018 for better comparison. The centers of mass of the FABP4 molecules in the two crystal forms are roughly at the same positions in the two lattices (Fig. 3 ▸), but the molecules differ by a rotation of ∼150° about an axis that is near-parallel to the *c* axis. For example, the superposition of 7fzw onto 7g0o has a direction cosine of (−0.016, −0.001, −0.999) through a point of fractional coordinates (0.284, 0.127, 0.219). Despite the axis being placed at nearly rational number 1/8 of the *b* axis, no relationship between the two settings could be detected using either the *CCP*4 program *OTHERCELL* or the Bilbao crystallographic server (Aroyo *et al.*, 2006 ▸), indicating that the similarity of the three axes in the two settings is likely fortuitous. In agreement with this, the two crystal forms display unique packing with different surface-located side chains engaging in the crystal contacts. Interestingly, all orthorhombic FABP4_3 structures were determined in space group 19, while all FABP4_5 structures belonged to space group 18 (‘3018’ in Table 1 ▸).

### 3.4. Bromine-containing ligands: phasing, radiation damage and σ-holes

**Figure 4:**
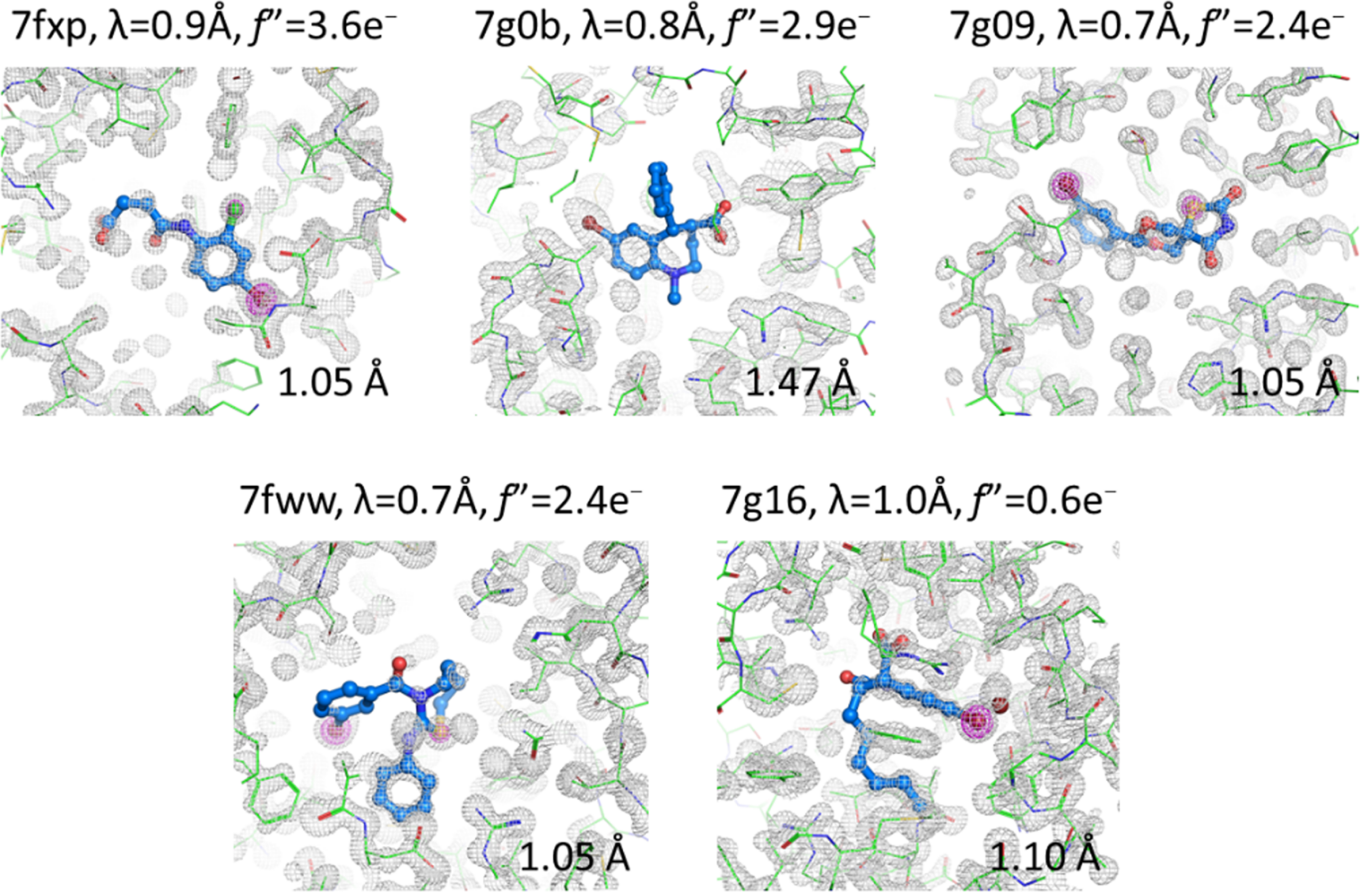
Five examples where SAD phasing based on bromine-containing ligands was successful. The SAD maps in gray are contoured with a radius of 10 Å at 1 r.m.s.d. around the ligand. SAD maps in magenta are contoured at 5 r.m.s.d. For the ligand in 7g0b, the Br atom did not display a signal beyond 5 r.m.s.d. For the structures 7fww and 7g09, a signal of >5 r.m.s.d. is displayed by the S atoms in the ligands. The final protein models are superimposed on the SAD maps. All structures are hFABP4 in space group 19, except 7g0b, which is hFABP5 in space group 96.

Nine data sets, seven from FABP4 in space group 19, one from FABP4_5 in space group 18 and one from FABP5 in the tetragonal space group 96, contained ligands with a Br atom (Table 2 ▸). Of these, despite the small calculated anomalous signal due to a suboptimal choice of incident X-ray wavelength, SAD phasing was successful in five cases (56%) and allowed an unbiased assessment of the ligand identities (Fig. 4 ▸). Four out of the five cases are isomorphous and in space group 19 with resolutions between 1.05 and 1.10 Å, multiplicities *m* of ∼6 and a calculated *f*′′(Br) between 0.6 and 3.6 e^−^. The fifth case is 7g0b in space group 96, which diffracted to a comparatively low resolution of 1.47 Å but due to the high crystal symmetry yielded a multiplicity of about 13 and a calculated *f*′′(Br) = 2.9 e^−^.

The smallest anomalous signal that still yielded SAD phases was *f*′′(Br) = 0.6 e^–^ at 1.0 Å wavelength in 7g16, which diffracted to 1.1 Å resolution in space group 19 with a multiplicity *m* of 5.8. By comparison, the best anomalous signal that did not yield SAD phases was *f*′′(Br) = 2.4 e^−^ for 7fym (FABP4_5) and 7fzw (FABP4) and comparable multiplicities of *m* = 5.4 and *m* = 6.5, respectively. These two data sets are at resolutions of 1.21 and 1.24 Å. In summary, in orthorhombic space groups high-resolution diffraction data of at least 1.1 Å resolution with a multiplicity of ∼6 together with *f*′′(Br) > 0.6 e^−^ are required for bromine SAD phasing of FABP. Lower resolution data such as in 7g0b (FABP5; space group 96, 1.47 Å resolution) require a higher multiplicity of at least 12.7 at comparable anomalous signal [λ = 0.8 Å, *i.e. f*′′(Br) = 2.9 e^−^] for successful phasing. High multiplicity, contributing to high accuracy of the intensities in the absence of radiation damage, has previously been shown to be important for sulfur SAD phasing (Doutch *et al.*, 2012 ▸; Weinert *et al.*, 2015 ▸), and this notion is of course valid for all situations with small anomalous signal present. Of note, an ultrahigh-resolution (0.88 Å) data set (7fxw) that contained only sulfur as a potential anomalous scatterer did not yield useful SAD phases, presumably because the data-collection wavelength of 0.7 Å ensured a too small *f*′′ of 0.12 e^−^, and the multiplicity was only *m* = 5.3. Using this data set as the native data in a SIRAS experiment on the failed Br-SAD cases did not solve the phase problem either.

**Figure 5:**
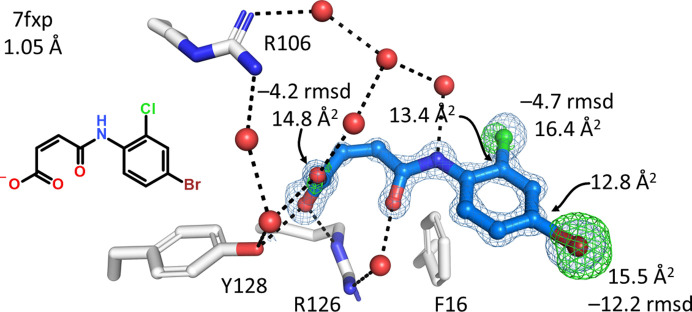
Radiation damage to the FABP4 ligand in 7fxp at 1.05 Å resolution. The negative maxima in r.m.s.d. of the *F*_o_ − *F*_c_ map after refinement are shown as green meshes at the carboxylate and the halogen atoms in the ligand, together with the *B* values of the atoms affected and their binding partners. Despite the damage to the Br atom, this data set retained enough anomalous signal for SAD phasing (see the first panel in Fig. 4 ▸). Refinement of the anomalous scattering factors did not change *f*′(Br) and only slightly changed *f*′′(Br) from 2.9 to 2.7 e^−^, with no effects on the *F*_o_ − *F*_c_ peak heights.

C—Br bonds, and also C—Cl bonds, S atoms and carboxylate groups, are prone to radiolysis by X-rays (Garman, 2010 ▸), and the question arises whether radiation damage might have contributed to the failure in SAD phasing of the structures 7fxa, 7fym, 7fzw and 7g0g (Table 2 ▸). A total of 22 FABP structures (9.6%) display strong negative *F*_o_ − *F*_c_ difference density peaks after refinement at the ligands, including all of the nine bromine-containing structures (Table 2 ▸). The presence of such peaks is indicative of radiation damage or incorrect anomalous dispersion correction during refinement. Of the structures affected, two are hFABP5, one is hFABP4_3, one is FABP4_5 and the rest are hFABP4. The resolution range covered in this subset is 0.95–1.47 Å and data were collected at wavelengths between 0.7 and 1.0 Å. Within this wavelength range, the anomalous scattering of bromine ranges from 0.6 to 2.9 e^−^. The results in the *F*_o_ − *F*_c_ maps were no different when the small anomalous scattering factors were specified during refinement (ANOM FORM BR keyword). As an example, in structure 7fxp, for which the data were collected at 0.8 Å wavelength, the estimated anomalous scattering factors *f*′/*f*′′ for bromine are −0.94/2.94 e^−^. Refinement against *F*/σ*F* without anomalous contribution and refinement against the separate Friedel pairs resulted in negligible differences in both *R*_free_ and residual *F*_o_ − *F*_c_ peaks at the Br atom of 16.6/16.8% and −8.5/−10.5 r.m.s.d., respectively. Consequently, incorrect anomalous dispersion correction could be ruled out as a source of the signals, leaving radiation damage as the culprit. A good example of the types of radiation damage visible in the ligands is 7fxp in complex with (*Z*)-4-(4-bromo-2-chloro­anilino)-4-oxobut-2-enoic acid. Three sites, the carboxylic acid and the halogen atoms, display negative *F*_o_ − *F*_c_ difference density peaks after refinement. The ligand was built at full occupancy, leading to elevated *B* values for the atoms that suffered radiolysis (Fig. 5 ▸). Despite the damage to the Br atom, this data set retained enough anomalous signal for SAD phasing (see the first panel in Fig. 4 ▸). Also, radiation damage is present at the bromine positions of the ligands in 7g09 (−15 r.m.s.d.), 7g0b (−6 r.m.s.d.), 7fww (−13 r.m.s.d.) and 7g16 (−7 r.m.s.d.) and also at the Cl positions of several other ligands (Supplementary Excel File S1). In structure 7g16, the radio­lyzed Br atom has relocated at van der Waals distance from the remaining ligand (bottom right panel in Fig. 4 ▸). In summary, radiation damage may have reduced the success rate of SAD phasing but has not led to the complete removal of anomalous signal from the data.

**Figure 6:**
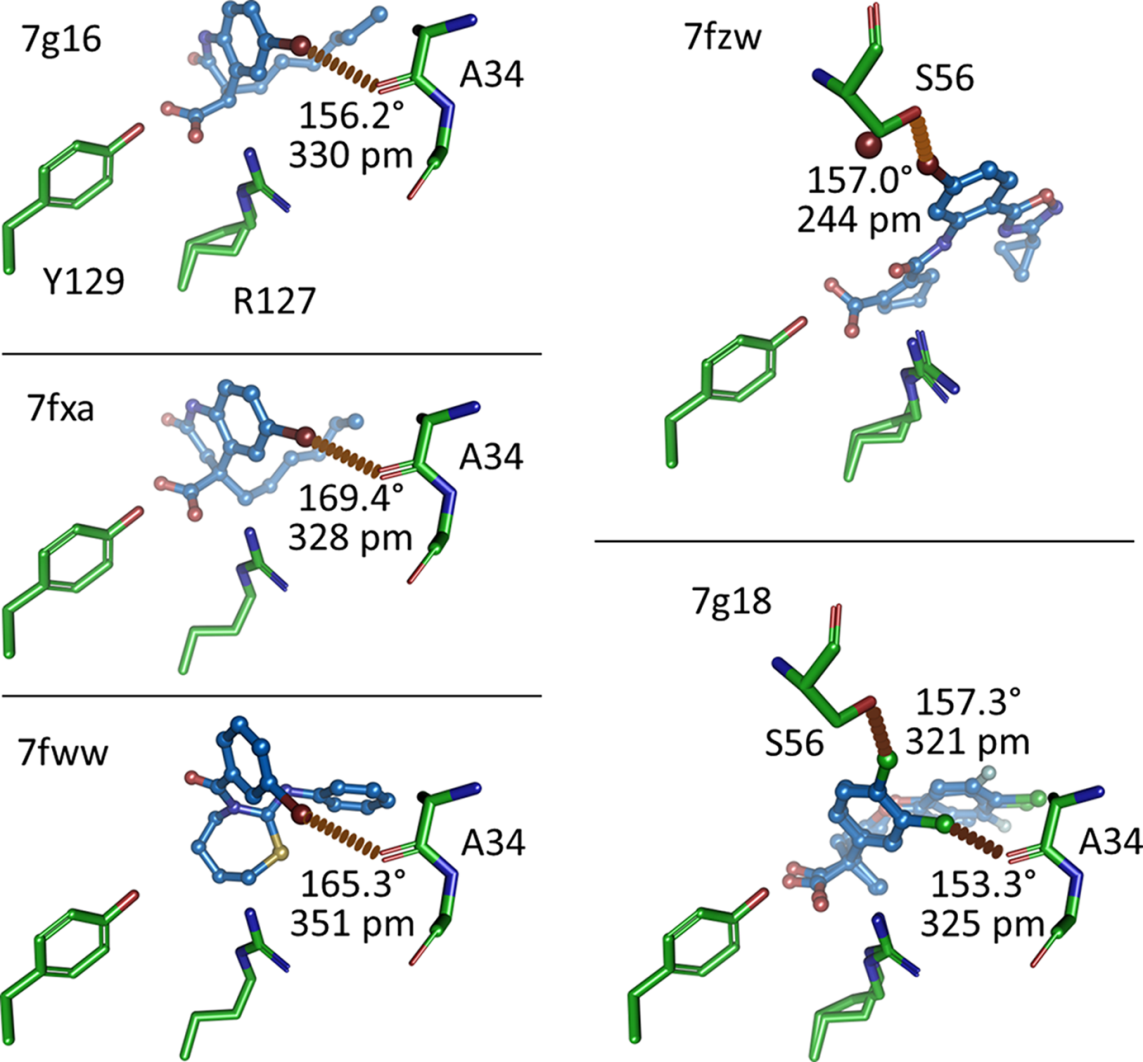
Halogen bonds in FABP ligands. The view is identical for all structures. Arg127 and Tyr129 are shown for reference and usually bind to the carboxylate group in the ligand. Note that the ligand in 7fww does not have a carboxylate group: it is neutral but still binds in a similar fashion to the ligands in 7g16 and 7fxa. The first three examples (7g16, 7fxa and 7fww) form a cluster of bromine halogen bonds to the carbonyl group of Ala34. 7fzw displays an unusually short bromine halogen bond, but this structure has also suffered radiation damage, with some of the liberated Br atoms (brown spheres) relocating to van der Waals distance to the O^γ^ atom of Ser56. This halogen bond should be interpreted with caution. The ligand in 7g18 exhibits two chlorine halogen bonds: one to the carbonyl group of Ala34 and one to the O^γ^ atom of Ser56.

Ligands containing C—*X* groups with *X* = Cl, Br, I can entertain halogen bonds, a noncovalent electrostatic inter­action between a nucleophile (*D*) and a positively charged patch at the tip of the C—*X* bond, termed the σ-hole (Clark *et al.*, 2007 ▸). The strength of the C—*X*⋯*D* bond increases with Cl < Br < I and also depends on both the distance between the halogen atom *X* and the nucleophile *D* and on the angle between the three atoms involved. Optimal energy and geometry requires a distance below the sum of the van der Waals radii of the interacting atoms and an angle of >140°, ideally 180°. Due to these rather strict geometric requirements, halogen bonds, similar to hydrogen bonds, are useful interactions for obtaining specificity in ligand design (Hardegger *et al.*, 2011*a* ▸,*b* ▸). In the FABP data set there is no ligand with an I atom, nine ligands contain a single Br atom and 79 ligands contain at least one Cl atom. Four of the nine bromine-containing ligands entertain a halogen bond with residues from FABP4 (Fig. 6 ▸). Three of them, 7g16, 7fxa and 7fww, form a cluster by halogen-bonding to the carbonyl group of Ala34. The C–Br vectors of the halogen bonds are slightly different, leading to a distance range between 321 pm (3.21 Å) and 351 pm (3.51 Å) and angles of between 156° and 169° to the carbonyl group of Ala34. The sum of the van der Waals radii of Br and O is 337 pm, indicating that the halogen bonds are not very short. An exception is made by 7fzw, which seems to engage in a very short halogen bond of only 244 pm with the side-chain O atom of Ser56. This bromine site has suffered radiation damage and some of the liberated Br atoms have shifted away from Ser56. Nevertheless, the remaining electron density for the ligand Br atom and, importantly, for the Ser56 residue is strong. Ser56 does not form alternate conformations in this structure, so the short halogen bond remains a conundrum, although bromine-mediated halogen bonds as short as 200 pm have been observed in small-molecule crystal structures (Anyfanti *et al.*, 2021 ▸). The other five structures, 7fxp, 7fym, 7g09, 7g0b and 7g0g, which also contain a Br atom in their ligands, do not display halogen bonds, but the Br atom engages in standard hydrophobic interactions. Turning to the case of chlorine, nine of the 79 chlorine-containing ligands display geometries in line with a halogen bond. Six of these (7fw7, 7fwb, 7fwq, 7fyb, 7fzs, 7fzt and 7g18) form a cluster to the carbonyl group of Ala34, indicating that Ala34 is a hotspot for such bonds in FABP. The distances and angles are in the ranges 321–357 pm and 153–172°. The sum of the van der Waals radii of Cl and O is 327 pm, again putting the distances of these halogen bonds at the long end. The Cl atoms in the other three structures form halogen bonds with O^γ1^ of Asp76 (7fxt; 304 pm and 168°), O^γ^ of Thr61 (hFABP4_5 structure 7g0c; 329 pm and 166°) and O^γ ^of Ser56 (7g18; 321 pm and 157°). The case of 7g18, which contains two chlorine halogen bonds, is shown in Fig. 6 ▸.

### 3.5. Ligand binding to FABP affects the lid and latch regions

**Figure 7:**
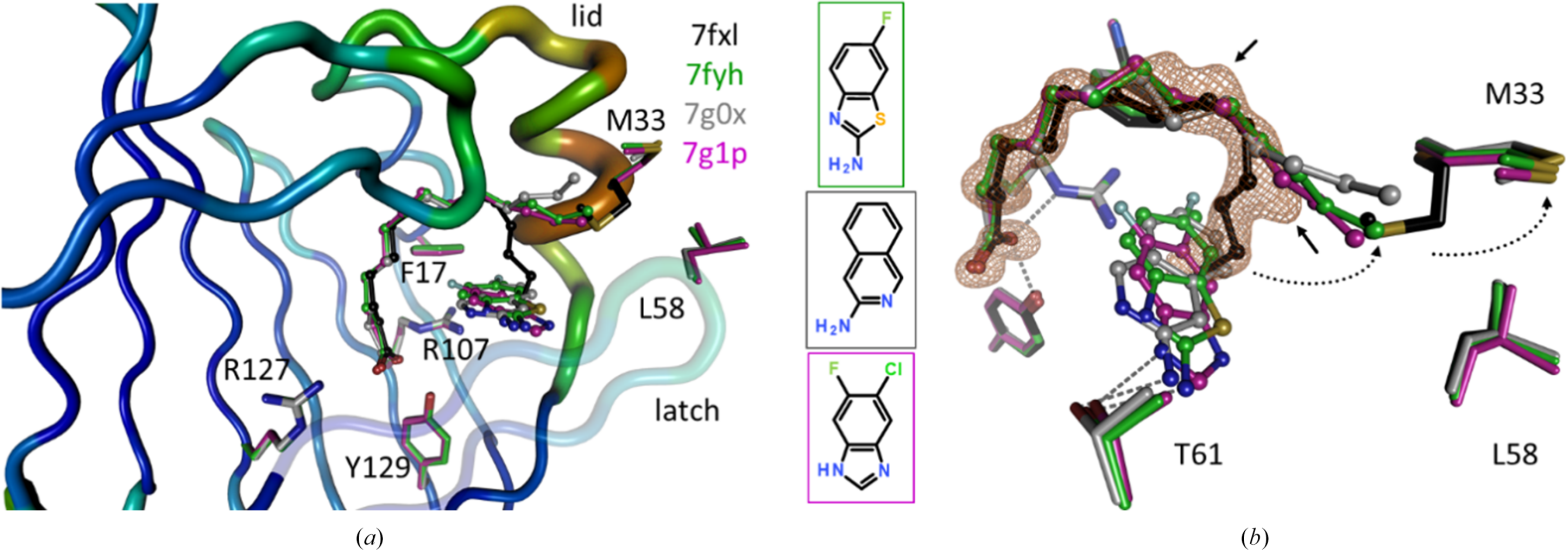
Joint fatty-acid and fragment binding to FABP4_5. (*a*) The backbone for hFABP4_5 structure 7fxl is shown as a *B*-value putty for reference, with the lid (Met33) and latch (Leu58) indicated. Ligands with an acidic head group, the carboxylate in fatty acids, bind between residues Arg107, Arg127 and Tyr129, often with charged hydrogen bonds. The aliphatic part of the fatty acids wraps around the tip of Phe17 to adopt U- or L-shaped conformations. For the four hFABP4_5 structures 7fxl, 7fyh, 7g0x and 7g1p, the endogenous fatty acid was built as myristate, but the presence of longer fatty acids as a mixture and multiple alternate conformations cannot be excluded [straight arrows in (*b*)]. 7fyh, 7g0x and 7g1p contain an additional fragment with the chemical structures shown. The hFABP4 structure 7fz8 also has a fragment bound at this site, but the density for the fatty acid was insufficiently clear to warrant its building. (*b*) All of the fragments engage in hydrogen bonding to the side chain of Thr61 and bind edge-on to the side chain of Phe17 (not shown). Fragment binding is incompatible with the U shape, but the tip of the aliphatic moiety shifts upwards and points into solvent. This shift is not accompanied by a change in the latch conformation or the Leu58 rotamer, but flips the side chain of Met33 in the lid (dotted arrows). The *F*_o_ − *F*_c_ omit electron density for the fatty acid in 7fxl is contoured as an orange mesh at the 3 r.m.s.d. level.

FABP isoforms bind to fatty acids of both different lengths and numbers of double bonds. Protein preparations therefore come with an array of endogenous fatty acids (see Casagrande *et al.*, 2025 ▸). In the absence of any displacing ligands, shorter fatty acids of ≤16 C atoms, including myristic and palmitic acids, and fatty acids of ≤20 C atoms with several *cis* double bonds, including linoleic and arachidonic acids, adopt a U shape centered at the tip of the conserved Phe17 side chain (hFABP4 UniProt numbering; Fig. 7 ▸). By contrast, longer fatty acids with a single *cis* double bond, such as oleic acid, adopt an L-shaped conformation with the end of the fatty acid contacting the lid and latch regions, ultimately pointing into bulk solvent.

The carboxylate group of fatty acids electrostatically interacts with the conserved side chains of Arg107, Arg127 and Tyr129, sometimes in the form of charged hydrogen bonds. In line with this observation and based on low *B* values, the carboxylate groups are quite fixed. In contrast, the aliphatic parts of the fatty acids display increasing *B* values towards the distal ends. The electron densities for fatty acids in the FABP data set invariably indicated flexibility of the aliphatic chain and often displayed alternate conformations (‘splitting’) at the ends. In all but one case (7fw5, for which palmitate could be built in the U conformation), myristate was the fatty acid best fitting the electron densities. The hydrophobic side chains lining the fatty acid-binding cavity seem to induce different conformations in the different FABP isoforms (see Casagrande *et al.*, 2025 ▸) but do not impose specificity for a particular fatty acid.

**Figure 8:**
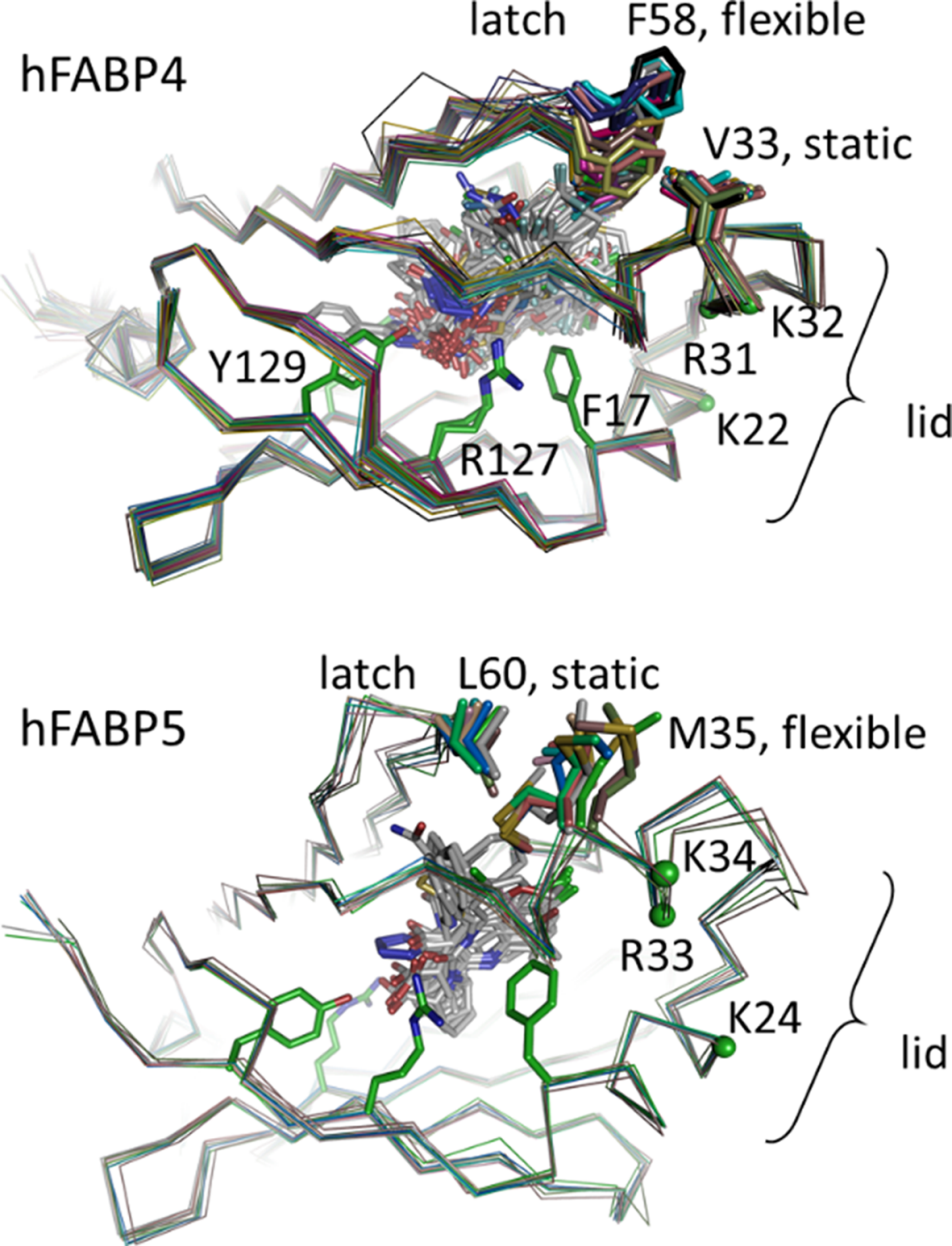
Inhibitor binding and its effect on lid and latch gating residues. All ligand-bound structures of hFABP4 (top) and hFABP5 (bottom) were superimposed and the conformations of the side chains of the gating residues were analyzed. In hFABP4 the gating residue Phe58 on the latch appears to be flexible, while the opposing gating residue Val33 on the lid retains its rotamer. In hFABP5 the situation is reversed, with the gating residue Leu60 on the latch appearing to be static and the gating residue Met35 on the lid being flexible. The side chains of Phe17, Arg127 and Tyr129 are shown as green stick models for reference. The three-dimensional non­classical NLS, Lys24, Arg33 and Lys34 in hFABP, is indicated by green spheres.

Soaking ligands at high concentrations usually displaced the fatty acids, except for the smallest fragments, which may co-bind to the FABP–fatty acid complex. We observed three such cases, all in complex with the construct hFABP4_5, which mimics the binding site of the FABP5 isoform within the framework of hFABP4 (Fig. 7 ▸). The ligands in 7fyh, 7g0x and 7g1p bind at the same site, interacting edge-on with the side chain of Phe17, and they form van der Waals contacts with the aliphatic part of the fatty acid. In addition, the three ligands have a hydrogen-bond donor that engages with the side chain of Thr61. Importantly, binding of the fragments is incompatible with the U-shaped fatty-acid conformation but induces the L shape. In all three cases the end of the fatty acid swings up towards the lid region, where it displaces the side chain of Met33 from an *in*- to an *out*-conformation. In contrast, the side chain of the latch residue Leu58 remains in place (Fig. 7 ▸). Given the space restraints inside the FABP ligand-binding cavity, similar conformational changes are likely to occur for all isoforms. Conformational changes in the lid and latch regions upon the binding of different ligands to FABP have previously been correlated with different biological activities (LiCata & Bernlohr, 1998 ▸; Gillilan *et al.*, 2007 ▸; Glaser *et al.*, 2023 ▸), including activation of a nonclassical nuclear import signal (NLS) in FABP4 (Ayers *et al.*, 2007 ▸). While medium-chain fatty acids of 12–16 C atoms are transported to mitochondria as metabolites, long-chain fatty acids of ≥18 C atoms induce translocation of FABP4 and FABP5 into the nucleus and promote gene expression (Xu *et al.*, 2022 ▸). Very long chain fatty acids bound to FABP5 activate nuclear receptors PPARβ/δ more strongly than do shorter chain fatty acids, leading to enhanced lipid-mediated gene expression affecting angiogenesis, cell proliferation and tumor progression (Furuhashi & Hotamisligil, 2008 ▸; Xu *et al.*, 2022 ▸). Taken together, this opens the interesting possibility that small, hydrophobic metabolites with similar size, shape and hydrogen-bonding capabilities to the examples in this FABP data set might co-bind to fatty acids and modulate their biological effects, at least in hFABP5. For hFABP5, it appears that Met33 in the lid region, not the latch residue Leu58, acts as a conformational sensor for ligand binding. We tested this hypothesis by separately superposing all available hFABP4 and hFABP5 structures to see which of the gating residues in the lid and latch regions exhibit the greatest plasticity (Fig. 8 ▸). Superposition of all ligand-containing hFABP4 structures (176) and all ten ligand-containing hFABP5 structures indeed shows differences in the effect of the ligands on the gating residues in the lid and latch regions. While for hFABP4 Phe58 in the latch region displays substantial plasticity, its hFABP5 counterpart Leu60 appears to be static. Conversely, while the hFABP4 lid residue Val33 that is closest to the ligand entry does not change its rotamer, its hFABP5 counterpart Met35 displays significant plasticity. This difference in the plasticity of the gating residues could thus indeed point to a different conformational sensor in the two FABP isoforms that reports on the bound ligand. The lid and latch regions communicate by way of the gating residues, which oppose each other and engage in van der Waals contacts, *i.e.* Val33/Phe58 in hFABP4 and Met35/Leu60 in hFABP5 (Fig. 8 ▸). In all structures, the lid and latch display elevated *B* values, and among the sets of structures these regions show increased r.m.s.d. values upon superposition. Also, the gating residues of these regions are not involved in crystal contacts but in fact often display alternate conformations. These observations indicate that movement of either gating residue affects the mobility of the other, effectively leading to the same result of conformational sensing. In hFABP4 the nonclassical NLS is composed of residues Lys22, Arg31 and Lys32 and these positions are conserved as Lys24, Arg33 and Lys34 in hFABP5 (Fig. 8 ▸). Since the nonclassical NLS is located in the lid region, a conformational sensing mechanism might allow decoding of the nature of the ligand into activation of nuclear transport. The frequent presence of alternate conformations of the mobile gating residues Phe58 (hFABP4) and Met35 (hFABP5) might be interpreted such that the signal delivered by the bound ligand to the gating residues is not an on/off switch but rather serves to modulate FABP biological activity, as exemplified by the increased gene-expression effects of fatty acids bound to FABP5 with increasing chain length (Furuhashi & Hotamisligil, 2008 ▸; Xu *et al.*, 2022 ▸). A similar three-dimensional signal of two lysine residues and one arginine residue at equivalent positions to hFABP4 and hFABP5 in the lid region has been proposed to be the nuclear translocation signal of FABP2 (Suárez *et al.*, 2020 ▸).

### 3.6. Acidic head groups

**Figure 9:**
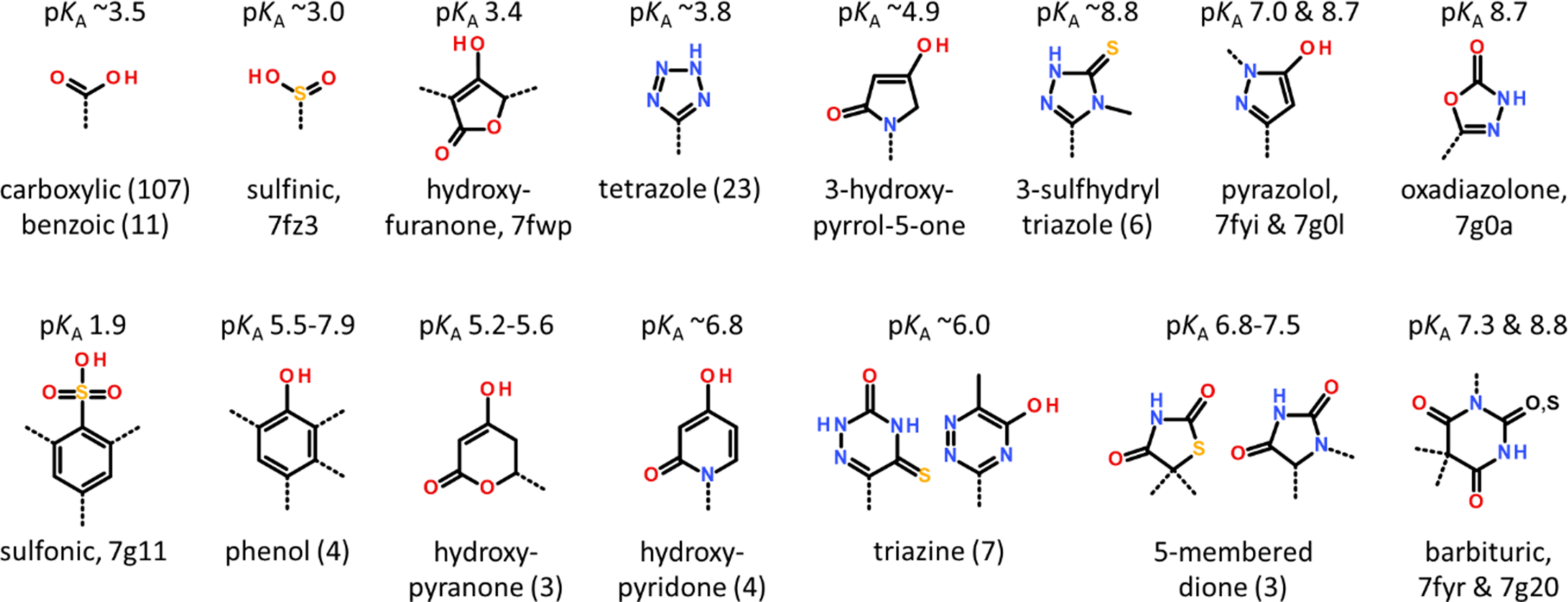
Acidic head groups in the FABP inhibitor set. The head groups are roughly sorted by size and increasing p*K*_a_ value. Exit vectors are noted by dashed lines and numbers in parentheses refer to the number of structures in that group, *e.g.* 11 benzoic acid derivatives. For ≤2 structures, *i.e.* the sulfinic and sulfonic acid, pyrazolols, oxadiazolone and barbituric acids, the PDB entries are given. The anticipated 3-hydroxy-pyrrol-5-one underwent oxidation, likely to the dihydroxy compound (7fxj).

The superposition of FABP structures shows that the ligands exhibit, in general, similar amphipathic properties and binding modes as fatty acids: a polar head group normally interacts with the conserved residues Arg107, Arg127 and/or Tyr129, and a more hydrophobic part interacts with residues that are usually in contact with the aliphatic part of a fatty acid (Fig. 8 ▸). Unsurprisingly, many of the screening hits contained an acidic head group (Fig. 9 ▸) and shared a similar binding mode, with the carboxylate contacting one or more of the residues Arg107, Arg127 and Tyr129 and a hydrophobic moiety packing edge-to-face against Phe17 (Figs. 4 ▸, 5 ▸ and 6 ▸). The structures of 107 carboxylic acid and 11 benzoic acid derivatives were determined. In addition, one sulfinic acid (7fz3) and one sulfonic acid (7g11) ligand was depicted in a crystal structure. Both the sulfonic acid and the sulfinic acid replace the carboxylic function. Of note, the sulfonic acid was either a hydrolysis product of or a starting material for an interesting screening hit, a sulfonyl-imidazole, that had no protolyzable group.

The low p*K*_a_ value of carboxylic acid and other acids of ≤3.5 makes them unsuitable for passive diffusion across membranes, so more attention was paid to mimetics of carboxylates with either larger p*K*_a_ values or where the negative charge resulting from protolysis is distributed by conjugation over more than three atoms (Fig. 9 ▸). Five- and six-membered heterocycles with a protolytic N atom in the ring or a hydroxy substituent bind similarly to FABP as do carboxylic acids, and many of these have p*K*_a_ values of >5, some near neutrality, that promise to allow a more facile membrane passage. Although many tetrazoles have low p*K*_a_ values of ∼4, this head group is an attractive and ‘almost isosteric’ carboxylic acid mimic that distributes the negative charge over the entire heterocycle. From the 23 crystal structures of tetrazoles that were determined, it is seen that compared with fatty acids the ligands are marginally pushed away from Arg127 and Tyr129 to fit the additional two atoms of the heterocycle into the protein. Compounds in this class could be developed with significant isoform specificity (Kühne *et al.*, 2016 ▸). For example, the same tetrazole ligand is present in 7fzy (hFABP4) and 5hz5 (hFABP4_5), displaying 68-fold and 16-fold higher activity against hFABP4 and hFABP5, respectively, compared with hFABP3. With the exception of the oxadiazolone in 7g0a discussed in Section 3.8, other small heterocycles such as pyrrolone, triazole and pyrazolols were less successful at inhibiting FABP and also sometimes less attractive synthetically. Larger head groups of six-membered rings mostly involved heterocycles with an acidic or enolic hydroxyl group such as phenol, hydroxypyridone, triazine or barbituric acid (Fig. 9 ▸). As hydroxyl groups are often subject to metabolic modification, such head groups were also de-prioritized.

### 3.7. Non-acidic head groups and peculiar binding modes

**Figure 10:**
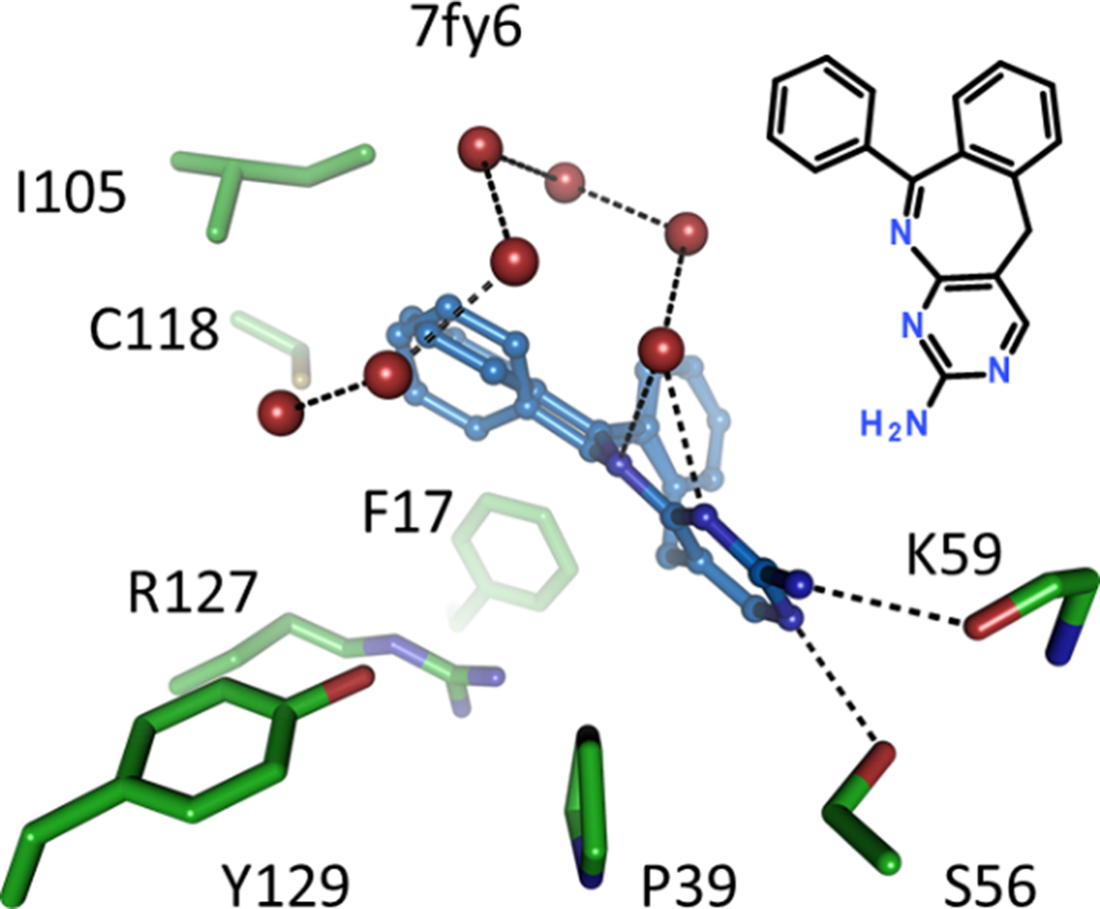
Non-acidic head group of a pyrimido-3-amine bound to hFABP4. No standard interactions with Arg07, Arg127 or Tyr129 are formed. Its binding involves the hydrophobic residues Phe17, Pro39, Ile105 and Cys118. Hydrogen bonds are formed with residues Ser56 and Lys59 in the latch region.

Nonprotolyzable head groups are rare in the FABP data set and such ligands either lack a chemical group where normally an acidic moiety is located, or they possess a group with a p*K*_a_ value that is >8 and thus is unlikely to dissociate significantly under physiological conditions. An example of a compound lacking a negative charge is found in 7fww, where the ligand has no carboxylate group at all but still binds with an IC_50_ of 1.8 µ*M* in a fashion similar to structurally related ligands that do contain a carboxylate group (Fig. 6 ▸). The oxadiazolone bound in structure 7g0a has a slightly basic head group with a calculated p*K*_a_ value of 8.7 but still binds in a similar pose (Fig. 13*b*) to that observed in negatively charged tetrazole-containing inhibitors such as that in 5hz5 (Kühne *et al.*, 2016 ▸). An outright basic compound with a calculated p*K*_a_ value of 9.8 is 6-phenyl-11H-pyrimido[4,5-c][2]benzazepin-3-amine in 7fy6 (Fig. 10 ▸). This molecule does not follow the usual interactions of a polar head group with the side chains of Arg107, Arg127 or Tyr129, but binds closer to the latch region of hFABP4 where the aminopyrimidine moiety can form hydrogen bonds to the side chain of Ser56 and the main-chain carbonyl group of Lys59. The rest of the molecule is hydrophobic and apart from the often-observed interaction with Phe17 also contacts Pro39, Ile105 and Cys118. A string of water molecules, connected by hydrogen bonds, is trapped around the hydrophobic parts of this ligand. Despite the absence of a negative charge or even a polar group in contact with arginine and tyrosine, the screening hit still binds with an appreciable IC_50_ for hFABP4 of 1.4 µ*M*, but was not developed further.

**Figure 11:**
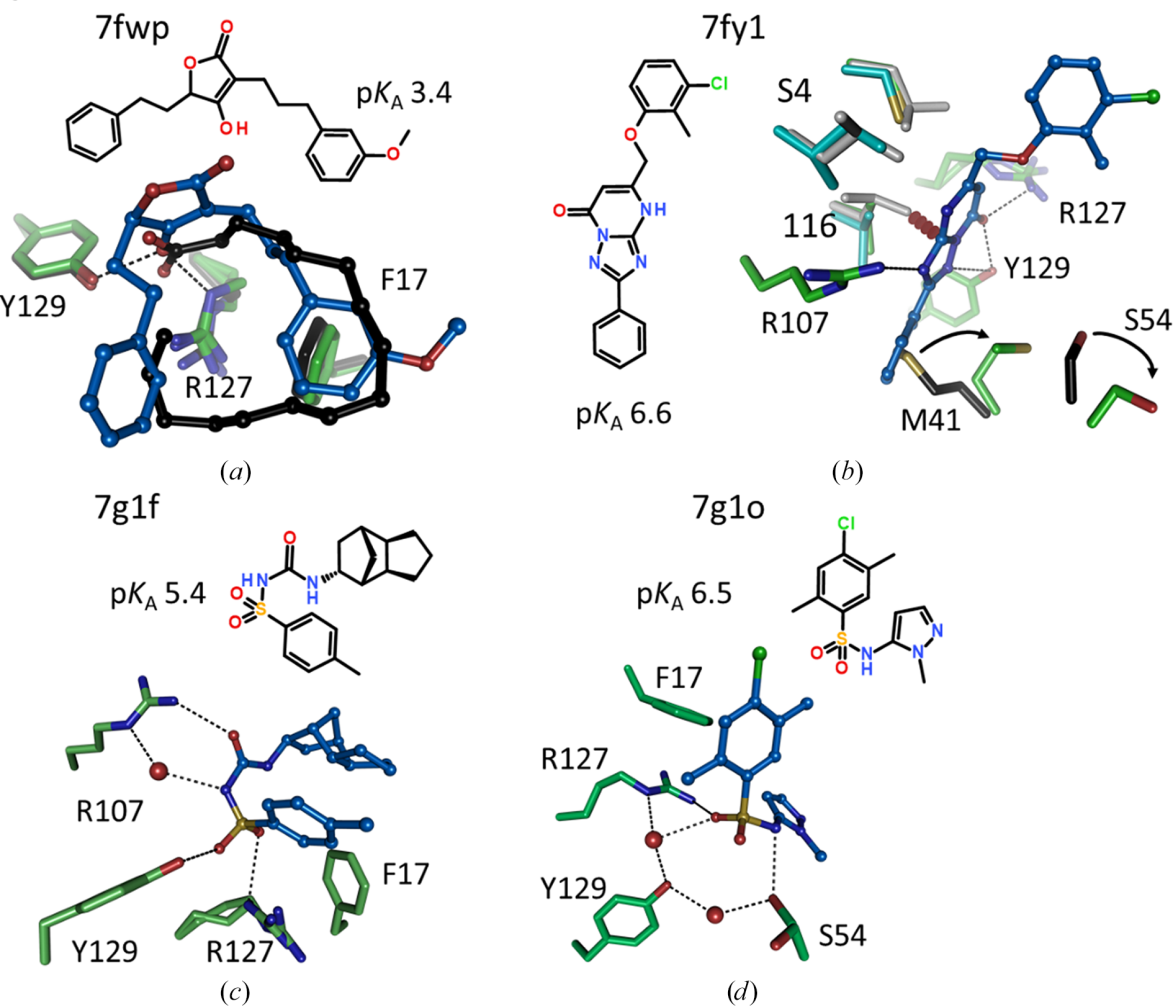
Nonstandard binding modes of acidic ligands in hFABP4. (*a*) The acidic moiety is at the center of the ligand in 7fwp. The ligand adopts a U shape and the hydrophobic interactions are similar to the palmitate in 2hnx (black), but the overall orientations of the ligands are different. (*b*) Binding mode of triazolo-pyrimidinone inhibitors. The isoforms hFABP3, hFABP4 and hFABP5 are colored gray, green and blue, respectively. The canonical orientations of the hFABP4 Met41 and Ser54 side chains are shown in black. The phenyl group attached to the triazole in this ligand class pushes Met41 into another preferred rotamer that requires Ser54 to also adopt another conformation (arrows). The p*K*_a_ value of the heterocycle is calculated as 6.6 and it forms three hydrogen bonds to Arg107, Arg127 and Tyr129. This compound is active on hFABP4 with an IC_50_ of 61 n*M*, but inhibits hFABP3 only weakly (IC_50_ of 8.7 µ*M*). The selectivity of this compound class against hFABP3 is ensured by stereochemical conflict with the side chain of Leu116 (red dashes). (*c*, *d*) The binding of the U-shaped sulfonamides is different from that of carboxylic acids and varies with compound. Some interactions with Arg107, Arg127 and Tyr129 are water-mediated.

Similar to 7fy6, some other ligands do not follow the general scheme of an elongated amphipathic molecule with a terminal acidic group and a hydrophobic tail occupying the space where fatty acids normally bind. These molecules, if they are negatively charged, either have their charge not terminally but in the middle of the otherwise hydrophobic molecule, or the head group is so large that the canonical binding mode cannot be formed. Such changes in overall binding modes include distancing of the ligand from the acid-binding side chains at the bottom of FABP and the opening of cryptic pockets. An example is the quite acidic hydroxy-furanone in 7fys, which has its acidic group in the middle of the molecule. While the conformation of the ligand is U-shaped and the space occupied by the ligand is similar to that of fatty acids, the exit vectors of the hydrophobic moieties from the polar head group are different such that the U shape is rotated compared with that of fatty acids (Fig. 11 ▸*a*).

A binding mode that opens a cryptic pocket is seen with triazolo-pyrimidinone inhibitors, originally developed by Merck (Lan *et al.*, 2011 ▸) and used in our study as reference compounds. Although the binding pose was described, no crystal structures were deposited in the PDB for this inhibitor class. A set of four crystal structures (7fyj, 7g06, 7fwa and 7fyt) consistently shows that the phenyl group attached to the triazole in this ligand class pushes Met41 into another preferred rotamer that requires Ser54 to also adopt another conformation (Fig. 11 ▸*b*). Such an opening of a cryptic pocket that allows the ligand to bind deeper within hFABP4 was not observed in any other of our ligand classes. The triazolo-pyrimidinone moiety is a weak acid with a calculated p*K*_a_ value of 6.6. The negative charge is in resonance over the ring surface and is neutralized by a set of hydrogen bonds to Arg107, Arg127 and Tyr129. Notably, the ligand is selective against hFABP3 (IC_50_ of 8.7 µ*M*) because Leu116 of the S4 pocket (see below) would clash with the heterocycle, while the corresponding Val116 in hFABP4 engages in attractive van der Waals interactions.

A structurally diverse set of five activated sulfonamide-containing ligands is found among the FABP data sets with p*K*_a_ values in the range 2.5–6.5. The sulfonamide is substituted with bulky hydrophobic groups that do not allow as close an approach of the head group to Arg107, Arg127 and Tyr129 as other ligands with smaller head groups do. Instead, water-mediated binding poses are present (Figs. 11 ▸*c* and 11 ▸*d*). Unless substituted with too stereochemically demanding groups, sulfonamides tend to adopt a U shape with an N—S torsion angle near zero. In the substituted sulfonamides in 7g1f and 7g1o these torsion angles are 67° and 77°, respectively, imposing an L shape for 7g1o, while 7g1f still adopts an overall U shape, not unlike that found for the U-shaped hydroxy-furanone in 7fwp (Fig. 11 ▸*a*).

### 3.8. Isoform specificity

**Figure 12:**
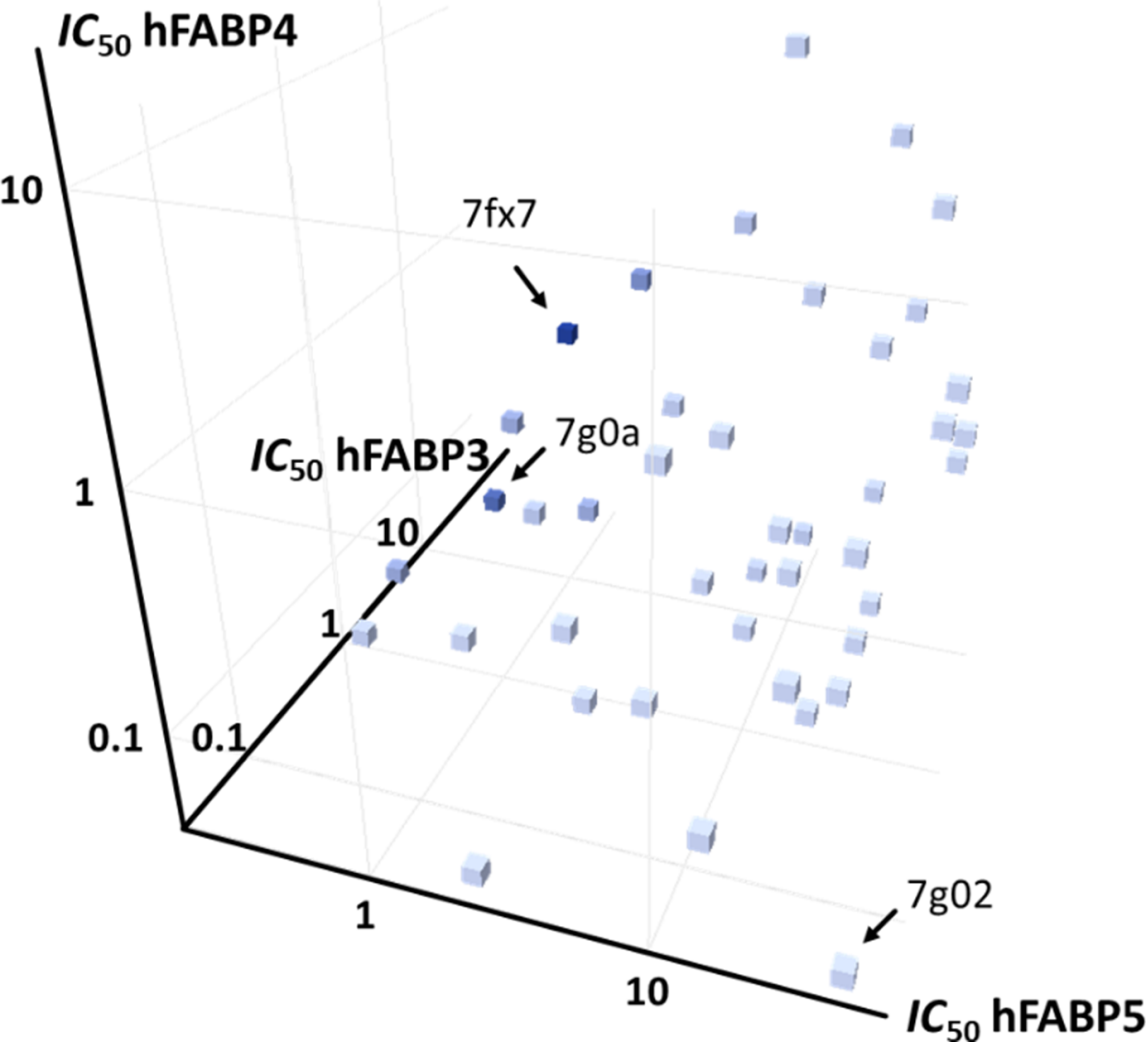
Isoform specificity. The IC_50_ values for 50 ligands where a crystal structure was also determined in one FABP isoform are plotted on logarithmic scales and colored in shades of blue according to the ratio IC_50__hFABP3/(IC_50__hFABP4 + IC_50__hFABP5). The darkest shades for 7fx7 and 7g0a correspond to the ligands with the best discrimination between hFABP3 and hFABP4/5. 7fx7 has a ratio of 44.1 and the ratio for 7g0a is 31.6. 7g02 is an example of a ligand that has dual activity on hFABP3 and hFABP4 but little activity on hFABP5.

The overall goal of FABP inhibitor development was an isoform-specific molecule that is active on both hFABP4 and hFABP5 but does not inhibit hFABP3. IC_50_ data on hFABP4 and hFABP5 are available for 76 ligands that are also depicted in crystal structures of at least one of the three isoforms (Supplementary Excel File S1). For a subset of 50 ligands, IC_50_ values for all three isoforms have been determined (Fig. 12 ▸), to which we limit the discussion here. A 3D graph of the IC_50_ values was prepared and colored according to the ratio IC_50__hFABP3/(IC_50__hFABP4 + IC_50__hFABP5), which should be large for high dual hFABP4/5 affinity and simultaneously high selectivity (*i.e.* low affinity) against hFABP3 (Fig. 12 ▸). The best discrimination between hFABP3 and hFABP4/5 in this set is achieved by the ligands in 7fx7 and 7g0a, for which the ratios are 44.1 and 31.6, respectively. 7fx7 has associated IC_50_ values of IC_50__hFABP3 = 45.08 µ*M*, IC_50__hFABP4 = 0.37 µ*M* and IC_50__hFABP5 = 0.65 µ*M* (selectivity window 69), while those for 7g0a are IC_50__hFABP3 = 14.43 µ*M*, IC_50__hFABP4 = 0.07 µ*M* and IC_50__hFABP5 = 0.39 µ*M* (selectivity window 37). Interestingly, there are also ligands in the set that display little discrimination against any of the three FABP isoforms or even an undesired pattern of dual hFABP3/4 inhibition with little activity on hFABP5. For example, 7g02 has IC_50__hFABP3 = 0.04 µ*M*, IC_50__hFABP4 = 0.04 µ*M* and IC_50__hFABP5 = 41.37 µ*M*.

**Figure 13:**
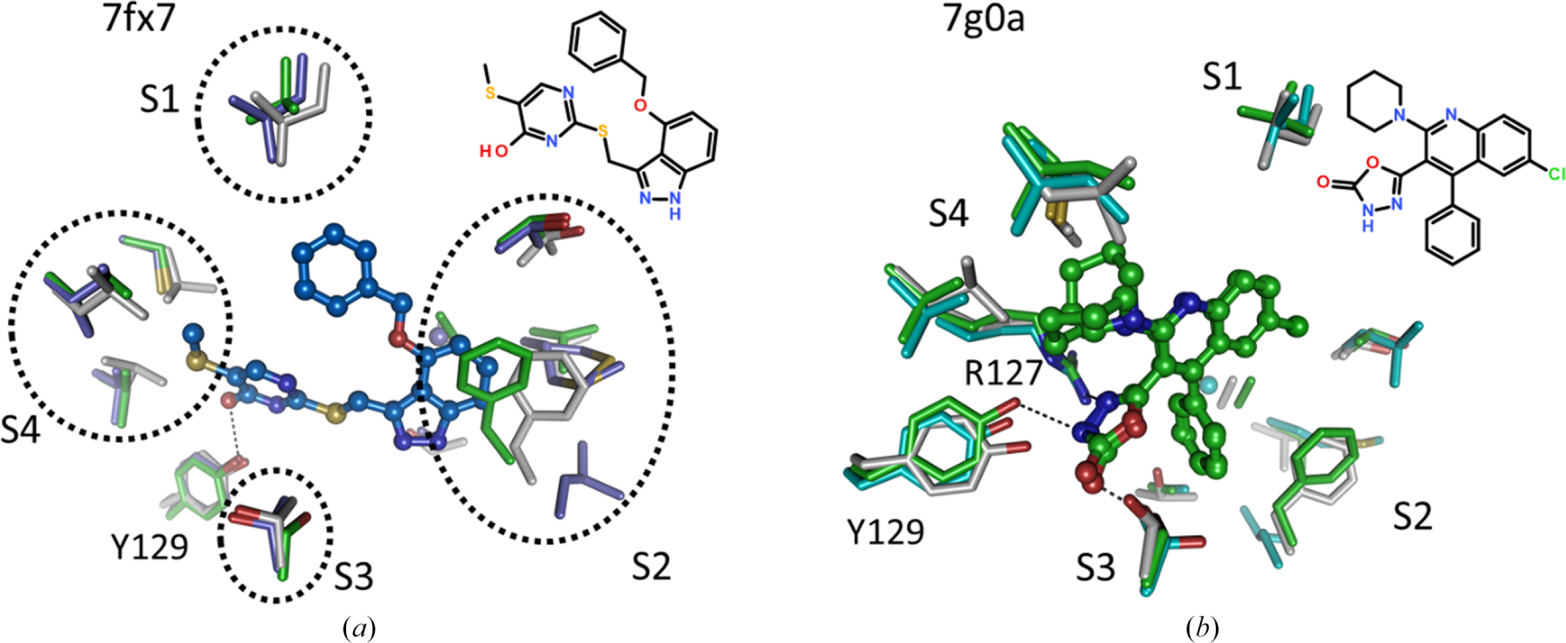
S4 is the main structural determinant for establishing isoform specificity. hFABP3, hFABP4 and hFABP5 are colored gray, green and blue, respectively. (*a*) Pockets with sequence and structural differences between the isoforms are labeled S1–S4 and enclosed by dashed ovals. 7fx7 is in complex with hFABP4_5, *i.e.* the binding site of hFABP5. The larger aliphatic side chains in S4 of hFABP3 would clash with the thiomethyl group of the ligand. (*b*) A similar discrimination against hFABP3 binding is apparent in 7g0a. Both alternate conformations of the ligand would clash with hFABP3 residues in S4.

A high-affinity (IC_50__hFABP4 = 0.05 µ*M* and IC_50__hFABP5 = 0.21 µ*M*) FABP4/5 dual inhibitor with a tetrazole head group (5hz5), significant discrimination against hFABP3 (ratio 12.9) and a favorable physicochemical profile has been described by us previously (Kühne *et al.*, 2016 ▸). In that study, by the superposition of the crystal structures of the three isoforms, we identified four possible regions, termed S1–S4, that could be exploited to install the desired isoform selectivity in ligands (Table 3 ▸). Pocket S1 contains just a single residue, Val24 in hFABP4, which is smaller than the equivalent positions of leucine in the other isoforms. The fact that the same residue is present in both hFABP3 and hFABP5 indicates this pocket to be unsuitable to generate dual FABP4/5 inhibitors while retaining selectivity against hFABP3. Pocket S2 is the structurally most versatile region, encompassing residues from the lid and latch regions. These display the largest conformational plasticity when all FABP structures are compared, indicating that this pocket might not be ideal for establishing selectivity. Like S1, the S3 pocket also has just a single residue, which is Ser54 in hFABP4 and threonine in the other isoforms, *i.e.* not a large difference in side-chain volume. Finally, the S4 pocket is identical in hFABP4/5 and has the largest differences from hFABP3, making it a promising region to establish the desired isoform selectivity. Indeed, for the ligand in 7fx7 determined in complex with hFABP4_5, superposition with hFABP4 and hFABP3 shows that the ligand will fit in hFABP4 but the residues in hFABP3 S4 will approach the ligand too closely. The three leucine residues at positions 105, 116 and 118 in hFABP3 would clash with the exocyclic thiomethyl group of the ligand, offering a stereochemical explanation for the poor affinity of the ligand against hFABP3 (Fig. 13 ▸*a*). This example also shows that pocket S2 is not well suited for incorporating selectivity in dual FABP4/5 inhibitors: Phe58 of the superposed hFABP4 structure occupies the same space as the ligand in hFABP4_5. Since the IC_50_ values for both isoforms are small, Phe58 must be able to undergo a conformational change to avoid clashing with the ligand. This change is indeed seen with other ligands reaching this area (Fig. 8 ▸). The second example is the ligand in 7g0a, determined in complex with hFABP4, where superposition with hFABP5 and hFABP3 also shows potential clashes of the ligand with side chains in S4 (Fig. 13 ▸*b*). In conclusion, for the task of isoform selectivity in hFABP4/5 against hFABP3, pocket S4 seems best-suited.

## 4. Conclusions and outlook

The set of FABP crystal structures encompasses the chemical space explored in the project over several years, highlighting several ways to replace the strongly acidic carboxylic acid moiety of fatty acids by almost isosteric and more drug-like groups. The structures together with the associated IC_50_ values could serve as a valuable resource to test, develop and improve methods for ligand-affinity prediction. As the data set unifies not a single target but structures and activity data on several isoforms, it should be suitable for studying isoform specificity *in silico* as well, which is a common task in many drug-design endeavors, including proteases, kinases and lipoxygenases, among others (Wang *et al.*, 2015 ▸; Rosenthal *et al.*, 2011 ▸; Kjer-Hansen *et al.*, 2024 ▸; Dana & Pathak, 2020 ▸; Aparoy *et al.*, 2012 ▸). The noncommercial PDBbind data set (version 2020) has 23 496 structures in complex with ligands that have experimentally determined affinity data (Wang *et al.*, 2004 ▸). This number could be increased for the development of more robust molecular-pose and affinity prediction tools. We would therefore like to take this opportunity to call on other industrial organizations to also make their legacy data available such that prediction models with broader applicability may be developed more quickly.

## 5. Related literature

The following reference is cited in the supporting information for this article: Henikoff & Henikoff (1992 ▸).

## Supplementary Material

PDB reference: human FABP4, complex with (*Z*)-4-(4-bromo-2-chloroanilino)-4-oxobut-2-enoic acid, 7fxp

Supplementary Excel File S1 detailing the crystal structures, ligands and IC50 values. DOI: 10.1107/S2059798325005728/gm5114sup1.xlsx

Supplementary Figure and Tables. DOI: 10.1107/S2059798325005728/gm5114sup2.pdf

## Figures and Tables

**Table 1:** Summary of FABP structures by crystal form For singletons in crystal forms, the PDB code is given in the first column. For the type of FABP in the second column, ‘h’ and ‘m’ refer to the human and mouse proteins, respectively. ‘4_3’ and ‘4_5’ are human FABP4 variants with key residues in the binding site changed to those in FABP3 and FABP5, respectively. Only unique cell constants are given, for example a single edge for 7fxd. Horizontal lines separate the FABP isoforms. Primitive orthorhombic systems are given with ascending cell constants for better comparison. The statistics include the six previously published FABP structures with PDB codes 5edb, 5edc, 5hz5, 5hz6, 5hz8 and 5hz9 (Kuhn *et al.*, 2016 ▸).

No.	PDB code	FABP	*n*	*a* (Å)	*b* (Å)	*c* (Å)	β (°)	Space group	Space group No.	Molecules in asymmetric unit	*d*_min_ (Å)
1	7g1x	h1	1	58.7	34.9	59.3	119.2	*P*2_1_	4	2	1.65
2	7fxo	h1	1	56.9	37.1	60.5	113.1	*P*2_1_	4	2	1.30
3	7fy8	h1	1	119.7	75.1	110.7	94.4	*C*2	5	8	2.23
4	7g0w	h1	1	38.9	106.7	56.3	90.4	*P*2_1_	4	4	1.64
5	7g00	h1	1	78.7	129.6	115.9	90.1	*P*2_1_	4	16	2.6
6	7fya	h1	1	38.7	56.4	111.0		*P*2_1_22_1_	2018	2	1.88
7	7fzq	h3	1	134.9	29.9	81.1	122.4	*C*2	5	2	1.60
8	5hz9	h3	1	186.8	186.8	114.2		*P*4_3_2_1_2	96	8	2.3
9		h4	3	137.1	137.1	137.6		*P*432	207	2	2.6, 2.6, 2.55
10	7fx3	h4	1	57.0		83.4		*P*4_3_2_1_2	96	1	1.12
11		h4	7	31.7–32.2	52.7–53.5	72.1–72.4		*P*22_1_2_1_	3018	1	1.12–1.74
12		h4_5	18	31.7–32.3	52.6–53.7	72.1–73.4		*P*22_1_2_1_	3018	1	0.84–1.64
13		h4	171	32.1–35.6	53.2–55.2	73.6–75.6		*P*2_1_2_1_2_1_	19	1	0.88–1.65, 1.96
14		h4_3	6	32.3–35.8	53.9–55.9	74.4–74.9		*P*2_1_2_1_2_1_	19	1	1.12–1.26
15	7fzk	h4_3	1	71.4	53.5	31.7	90.4	*C*2	5	1	1.12
16	7fwt	m4	1	50.0	78.0	94.5		*P*2_1_2_1_2_1_	19	2	1.54
17	7fzg	m4	1	77.8	94.7	49.9		*C*222_1_	20	1	1.49
18		h5	7	61.9–63.1		74.5–75.7		*P*4_3_2_1_2	96	1	1.11–1.55
19	7g04	h5	1	61.4	62.9	74.4		*P*2_1_2_1_2_1_	19	2	1.40
20	7fwi	h5	1	54.4	38.4	80.3		*P*2_1_	4	3	2.0
21	7fxd	h5	1	154.5				*F*432	209	1	2.44
22	7fyw	m5	1	59.6	75.1	61.8	90.2	*P*2_1_	4	4	1.81
23	7fy1	h9	1	108.0		76.9		*P*6_5_22	179	1	2.06

**Table 2:** SAD phasing by ligands CC, correlation coefficient in %. φ, phases. All data sets have a single FABP molecule per asymmetric unit with high resolution *d*_max_, multiplicity *m* and data-collection wavelength λ. 1000 *SHELXD* trials were run, followed by 200 cycles of density modification in *SHELXE* with 50% solvent content and ten cycles of rebuilding without searching for secondary structures. Note the unusually small CC_all_/CC_weak_ of <10% for 7g16 that still yielded phases. For φ labeled ‘—’ in *P*2_1_2_1_2_1_, both SAD and SIRAS using 7fxv as the native data set were tried, without success. The starting data were XDS_ASCII.HKL. Other scaling regimes (*i.e.AIMLESS* with *STARANISO* anisotropy analyses) did not yield different results. The last column labeled ‘r.m.s.d.’ shows the largest positive and negative peak heights of a difference map calculated using the measured anomalous differences and the refined phases, where ‘ok’ and ‘no’ indicate whether the largest positive peak corresponds to the strongest anomalous scatterer.

PDB code	FABP	Space group No.	*d*_max_ (Å)	*m*	λ (Å)	Element	*f*′′ (e^−^)	CC_all/weak_	φ	CC_part_	No. of residues	r.m.s.d.
7fxv	4	19	0.88	6.5	0.7	S	0.1	7.4/2.9	—	n.a.	n.a.	5.4/−5.2, no
7fww	4	19	1.05	6.5	0.7	Br, S	2.4, 0.1	21.4/14.3	SAD	45	134	11.0/−5.1, ok
7fxa	4	19	1.12	6.2	1.0	Br, S	0.6, 0.2	4.7/2.0	—	n.a.	n.a.	7.7/−8.3, ok
7fxp	4	19	1.05	6.4	0.8	Br, Cl, S	2.9, 0.2, 0.2	18.0/12.2	SAD	27	133	12.5/−10.9, ok
7fym	4_5	18	1.21	5.4	0.7	Br, S	2.4, 0.1	6.8/4.2	—	n.a.	n.a.	7.1/−5.2, ok
7fzw	4	19	1.24	6.5	0.7	Br, S	2.4, 0.1	7.4/4.5	—	n.a.	n.a.	5.0/−5.0, no
7g09	4	19	1.05	6.1	0.7	Br, S	2.4, 0.1	16.4/11.4	SAD	26	136	8.7/−5.0, ok
7g0b	5	96	1.47	12.7	0.8	Br, S	2.9, 0.2	17.1/10.2	SAD	33	124	4.9/−4.6, no
7g0g	4	19	1.12	6.1	1.0	Br, S	0.6, 0.2	4.6/2.5	—	n.a.	n.a.	4.5/−6.5, no
7g16	4	19	1.10	5.8	1.0	Br, S	0.6, 0.2	8.9/5.9	SAD	44	134	10.9/−4.7

**Table 3:** Sequence differences in the ligand-binding site between isoforms hFABP3/4/5 Residues that are unique to a single isoform are in bold. The residues in selectivity pocket S2 are the gating residues in the lid and latch regions of FABP4.

	hFABP3	hFABP4	hFABP5
S1	Leu24	**Val24**	Leu26
S2	Thr30, Val33, Ala34, **Thr37**, Phe58	Thr30, Val33, Ala34, Ala37, Phe58	**Leu32**, **Met35**, **Gly36**, Ala39, **Leu60**
S3	Thr54	**Ser54**	Thr56
S4	**Leu105**, **Leu116**, **Leu118**	Ile105, Val116, Cys118	Ile107, Val118, Cys120

## Data Availability

The structures reported in this article have been deposited in the Protein Data Bank (https://www.rcsb.org) with Group Deposit ID G_1002264.
