# Supplementary material for: A high-resolution data set of fatty acid-binding protein structures. II. Crystallographic overview, ligand classes and binding pose
Source: Acta Crystallogr D Struct Biol. 2025 Jul 28;81(Pt 8):436–50. doi: 10.1107/S2059798325005728 (PMC12315584; doi:10.1107/S2059798325005728)
Supplement: Supplementary file 2 [file d-81-00436-sup2.pdf]

STRUCTURAL
BIOLOGY

Volume 81 (2025)

Supporting information for article:

A high-resolution data set of fatty acid-binding protein structures. II: Crystallographic overview, ligand classes and binding pose

Andreas Ehler, Joerg Benz and Markus G. Rudolph

	1							58	
hFABP4	GSHMC..DAF	VGTWKLVSSE	NFDDYMKEVG	VGFATRKVAG	MAKPNMIISV	NGDVITIKSE	**S**T**F**KNTEIS	64
hFABP4_3	GSHMC..DAF	VGTWKLVSSE	NFDDYMKELG	VGFATRKVAG	MAKPNTIISV	NGDVITLKTE	**S**TFKNTEIS	64
hFABP4_5	GSHMC..DAF	VGTWKLVSSE	NFDDYMKELG	VGFATRKMGG	MAKPNCIISV	NGDVITIKTE	**S**TLKNTEIS	64
hFABP3	GSHMV..DAF	LGTWKLVDSK	NFDDYMKSLG	VGFATRQVAS	MTKPTTIIEK	NGDILTLKTH	STFKNTEIS	64
hFABP5	GSHMATVQQL	EGRWRLVDSK	GFDEYMKELG	VGIALRKMGA	MAKPDCIITC	DGKNLTIKTE	STLKTTQFS	66
hFABP9	GSHMV..EPF	LGTWKLVSSE	NFEDYMKELG	VNFAARNMAG	LVKPTVTISV	DGKMMTIRTE	SSFQDTKIS	64
hFABP1	GSHMS....F	SGKYQLQSQE	NFEAFMKAIG	LPEELIQKGK	DIKGVSEIVQ	NGKHFKFTIT	AGSKVIQNE	62
mFABP4	GSHMC..DAF	VGTWKLVSSE	NFDDYMKEVG	VGFATRKVAG	MAKPNMIISV	NGDLVTIRSE	STFKNTEIS	64
mFABP5	GSHMASLKDL	EGKWRLMESH	GFEEYMKELG	VGLALRKMAA	MAKPDCIITC	DGNNITVKTE	STVKTTVFS	66

hFABP4	FILGQEFDEV	TADDRKVKST	ITLDGGVLVH	VQKWDGKSTT	IK**R**KREDDKL	VVECVMKGVT	ST**R**V**Y**ERA	132
hFABP4_3	FILGQEFDEV	TADDRKVKST	ITLDGGVLVH	VQKWDGKSTT	LK**R**KREDDKL	VLELVMKGVT	CT**R**V**Y**ERA	132
hFABP4_5	FILGQEFDEV	TADDRKVKST	ITLDGGVLVQ	VQKWDGKSTT	IK**R**KREDDKL	VVECVMKGVT	CT**R**V**Y**ERA	132
hFABP3	FKLGVEFDET	TADDRKVKSI	VTLDGGKLVH	LQKWDGQETT	LVRELIDGKL	ILTLTHGTAV	CTRTYEKEA	132
hFABP5	CTLGEKFEET	TADGRKTQTV	CNFTDGALVQ	HQEWDGKEST	ITRKLKDGKL	VVECVMNNVT	CTRIYEKVE	135
hFABP9	FKLGEEFDET	TADNRKVKST	ITLENGSMIH	VQKWLGKETT	IKRKIVDEKM	VVECKMNNIV	STRIYEKV	132
hFABP1	FTVGEECELE	TMTGEKVKTV	VQLEGDNKLV	TTFKNIKS..	.VTELNGDII	TNTMTLGDIV	FKRISKRI	127
mFABP4	FKLGVEFDEI	TADDRKVKSI	ITLDGGALVQ	VQKWDGKSTT	IKRKRDGDKL	VVECVMKGVT	STRVYERA	132
mFABP5	CNLGEKFDET	TADGRKTETV	CTFQDGALVQ	HQQWDGKEST	ITRKLKDGKM	IVECVMNNAT	CTRVYEKVQ	135

Figure S1 Alignment of FABP isoforms discussed here. Label "1" denotes start of the protein, the sequence GSH is a cloning artifact. Residues frequently interacting with ligands are in boldface. Residues changed from FABP4 to match the binding site of FABP3 and FABP5 are underlined. FABP4_3 is FABP4 variant V24L, M41T, I52L, S54T, I105L, V116L, C118L, S125C. FABP4_5 is FABP4 variant V24L, V33M, A34G, M41C, S54T, F58L, H94Q, S125C, with FABP4 numbering.

Table S1 Pairwise sequence alignments of FABP4 isoforms

isoform	h3	h4	h4_3	h4_5	h5	h9	m4	m5
h1	29.8/45.8	24.3/40.4	24.8/45.0	26.2/43.4	23.5/43.9	25.6/45.0	24.8/44.0	25.7/41.6
h3		**64.9/77.1**	71.0/79.4	64.1/75.6	**51.2/67.2**	55.7/77.1	64.9/77.9	50.4/68.8
h4			93.9/97.7	93.9/96.2	**55.2/72.0**	64.1/84.0	**91.7/95.5**	54.4/72.0
h4_3				93.2/96.2	54.4/72.0	61.8/81.7	85.6/93.2	54.4/72.0
h4_5					61.6/76.0	63.4/80.2	87.1/93.2	58.4/74.4
h5						51.1/70.2	55.2/72.0	**80.0/93.3**
h9							63.4/82.4	52.4/70.6
m4								**53.6/72.0**

Numbers are identity/similarity percentages as defined in the BLOSUM62 matrix (Henikoff & Henikoff, 1992). "h" and "m" are for human and mouse proteins. The isoforms 1, 3, 4, 5, and 9 are also known as liver-, heart-, adipocyte-, epidermal-, and testis-FABP. Other isoforms not discussed here are FABP2 (intestinal), FABP6 (ileum), FABP7 (brain), FABP8 (myelin), and FABP12. Human-human and mouse-mouse comparisons are highlighted in bold black and bold blue, respectively. Inter-species comparisons of particular interest are highlighted in orange.

Table S2 Crystallization conditions

#	ID	FABP	SG	reservoir	cryo
1	7g1x	h1	$P2_1$	30% P2K MME, 0.15 M benzamidine	15% EG
2	7fxo	h1	$P2_1$	30% P2K MME, 0.15 M benzamidine	15% EG
3	7fy8	h1	C121	30% P2K MME, 0.1 M KSCN	15% EG
4	7g0w	h1	$P2_1$	36% P2K MME, 2% benzamidine	–
5	7g00	h1	$P2_1$	30% P2K MME, 0.15 M benzamidine, 0.1 M KSCN	20% EG
6	7fya	h1	$P2_122_1$	36% P2K MME, 2% benzamidine	–
7	7fzq	h3	C2	20% PEG 3350, 0.2 M $LiNO_3$ pH 7.1 (PEG/ion screen B3)	20% EG
8	5hz9	h3	$P4_32_12$	22.5% PEG 8K, 0.1 M NaOAc pH 5, 0.2 M Li_2SO_4	20% EG
9		h4	P432	2.4 M $(NH_4)_2SO_4$, 0.1 M HEPES/NaOH pH 8	oil
10	7fx3	h4	$P4_32_12$	25% PEG 3350, 0.2 M NaCl, 0.1 M Tris/HCl pH 8.5	15% EG
11		h4	$P22_12_1$	30% PEG 3350, 0.2 M Li_2SO_4, 0.1 M bis-Tris/HCl pH 6.5	–
12		h4_5	$P22_12_1$	30% PEG 3350, 0.2 M Li_2SO_4, 0.1 M bis-Tris/HCl pH 6.5	–
13		h4	$P2_12_12_1$	30% PEG 3350, 0.2 M Li_2SO_4, 0.1 M bis-Tris/HCl pH 6.5	20% EG
14		h4_3	$P2_12_12_1$	30% PEG 3350, 0.2 M Li_2SO_4, 0.1 M bis-Tris/HCl pH 6.5	20% EG
15	7fzk	h4_3	C2	25% PEG 3350, 0.2 M $MgCl_2$, 0.1 M bis-Tris/HCl pH 5.5 (Index G10)	15% EG
16	7fwt	m4	$P2_12_12_1$	25% PEG 3350, 0.1 M citric acid pH 3.5 (Index D4)	20% EG
17	7fzg	m4	$C222_1$	2.4 M sodium malonate pH 7.0 (Index C3)	oil
18		h5	$P4_32_12$	45% PEG 550 MME, 0.15 M Li_2SO_4, 0.15 M Tris/HCl pH 7.5	–
19	7g04	h5	$P2_12_12_1$	37% Peg 550 MME, 0.1 M Li_2SO_4, 0.02 M citric acid, 0.1 M Tris/HCl pH 7.5	–
20	7fwi	h5	$P2_1$	30% P2K MME, 0.15 M KBr (Index H12)	15% EG
21	7fxd	h5	F432	1.4 M Sodium citrate, 0.1 M HEPES/NaOH pH 7.5 (Index B8)	oil
22	7fyw	m5	$P2_1$	30% P2K MME, 0.1 M KSCN (Index H11)	20% EG
23	7fy1	h9	$P6_522$	1.8 M ammonium citrate pH 7.0 (Index B9)	oil

EG: ethylene glycol; PEG: polyethylene glycol; MME: monomethyl ether. “oil” is paraffin oil. PEG/ion and Index are the screens from Hampton.

Table S3 Halogen bonds identified in FABP structures

structure	halogen	contact	length (pm)	angle (°)
7g16	Br	Ala34 Oγ	330	156.2
7fxa	Br	Ala34 Oγ	328	169.4
7fww	Br	Ala34 Oγ	351	165.3
7fzw	Br	Ser56 Oγ	244	157.0
7fwt	Cl	Ala34 Oγ	333	165.9
7fwb	Cl	Ala34 Oγ	338	172.6
7fwq (43)	Cl	Ala34 Oγ	340	168.6
7fxt	Cl	Asp76 Oγ1	304	168.3
7fyb	Cl	Ala34 Oγ	321	176.8
7fzs	Cl	Ala34 Oγ	357	167.5
7fzt (45)	Cl	Ala34 Oγ	330	166.3
7g0c (45)	Cl	Ala34 Oγ	329	165.6
7g18	Cl	Ala34 Oγ	325	153.3
	Cl	Ser56 Oγ	321	157.3

Numbers (43) and (45) are constructs FABP4_3 and FABP4_5, respectively, all other examples are from wild-type FABP4.
